# Supplementary material for: Impact of selective licensing schemes for private rental housing on mental health and social outcomes in Greater London, England: a natural experiment study
Source: BMJ Open. 2022 Dec 23;12(12):e065747. doi: 10.1136/bmjopen-2022-065747 (PMC9791445; doi:10.1136/bmjopen-2022-065747)
Supplement: Supplementary data [file bmjopen-2022-065747supp001.pdf]

Supplementary materials

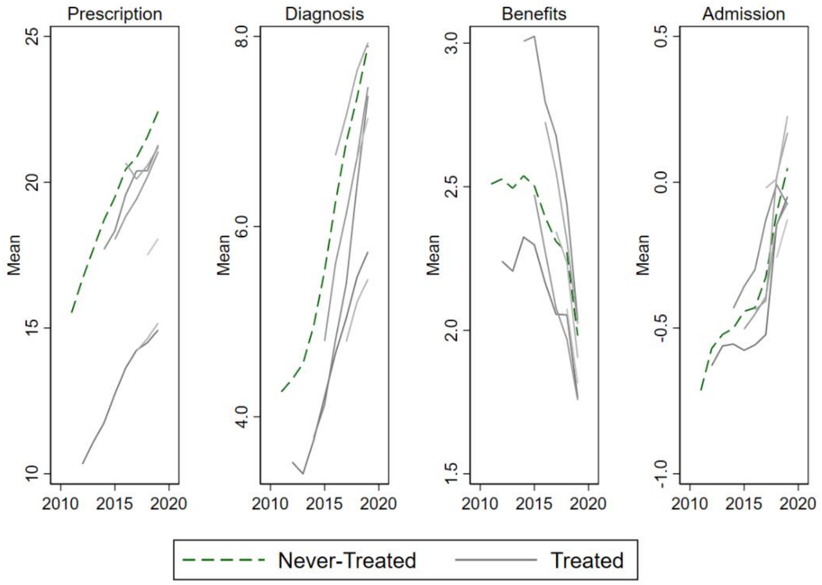

Supplementary Figure 1 Trend in Small Area Mental Health Index (SAMHI) sub-scores (PRESCRIPTION, DIAGNOSIS, BENEFITS, ADMISSION) for never-treated versus treated areas in Greater London, 2011-2019. Treated areas shown from year of initiation onwards..

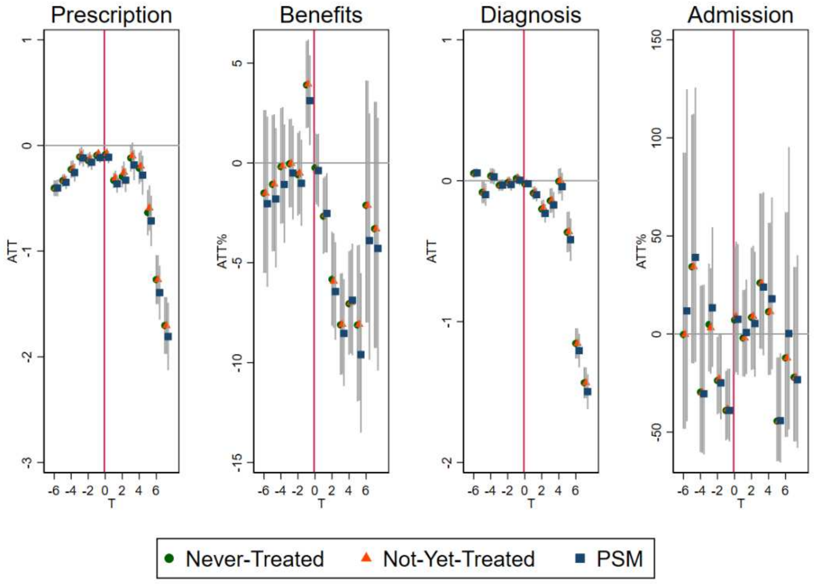

Supplementary Figure 2 Average treatment effect on the treated (ATT) for area-level impacts of selective licencing (SL) on Small Area Mental Health Index (SAMHI) underlying indicators PRESCRIPTION, BENEFITS, DIAGNOSIS, and ADMISSION, in Greater London, 2011-2019. BENEFITS and ADMISSION were ln-transformed and ATT shown as ATT%.

Petersen J, *et al. BMJ Open* 2022; 12:e065747. doi: 10.1136/bmjopen-2022-065747

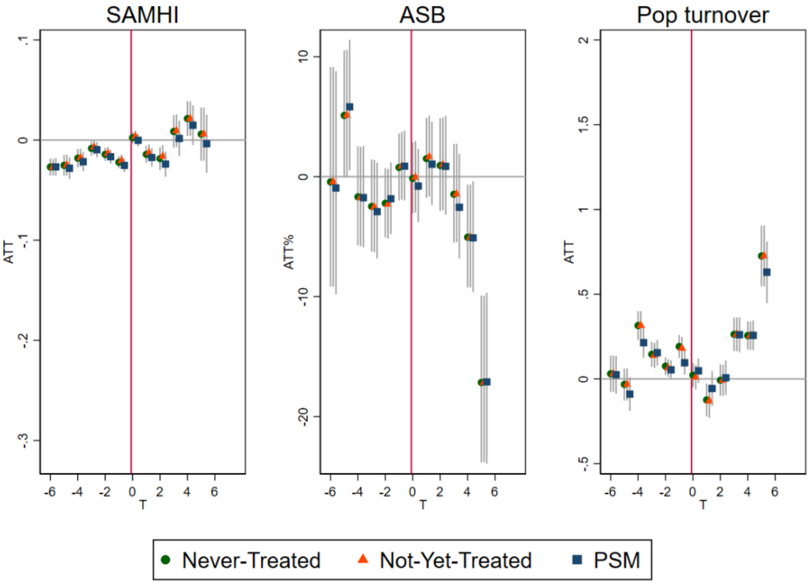

Supplementary Figure 3 Sensitivity check excluding the earliest scheme initiated 2012 ("Olympic"). Average treatment effect on the treated (ATT) for area-level impacts of selective licencing (SL) on Small Area Mental Health Index (SAMHI), Antisocial behaviour (ASB) calls, and population (Pop) turnover in Greater London, 2011-2019. ASB was ln-transformed and ATT shown as ATT%.

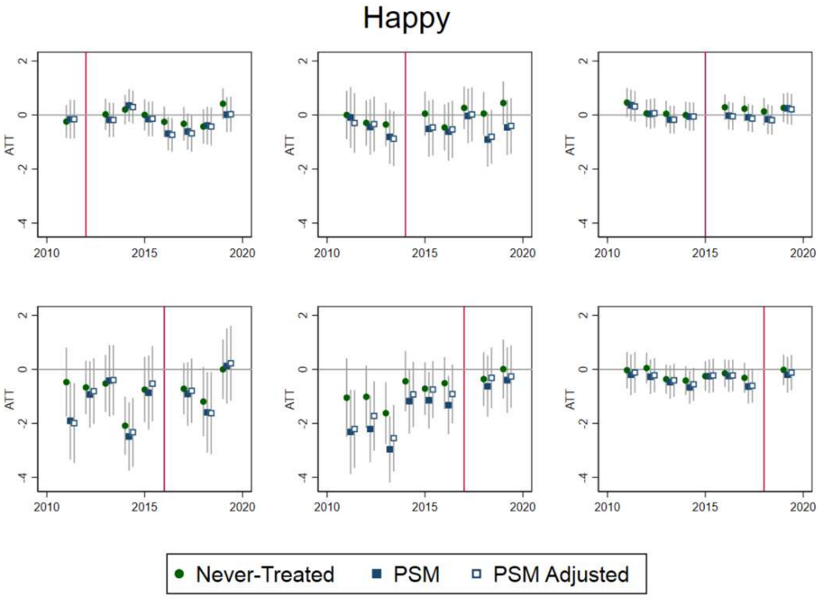

Supplementary Figure 4 Happy. Average treatment effect on the treated (ATT) for individual-level impacts of selective licencing (SL) on self-reported happy score among private renters in Greater London by year of SL introduction, 2011-2019. Time-varying covariates in PSM Adjusted were: age group, sex, native birth, and occupational class. Abbreviations: Propensity Score Matching (PSM).

Petersen J, *et al. BMJ Open* 2022; 12:e065747. doi: 10.1136/bmjopen-2022-065747

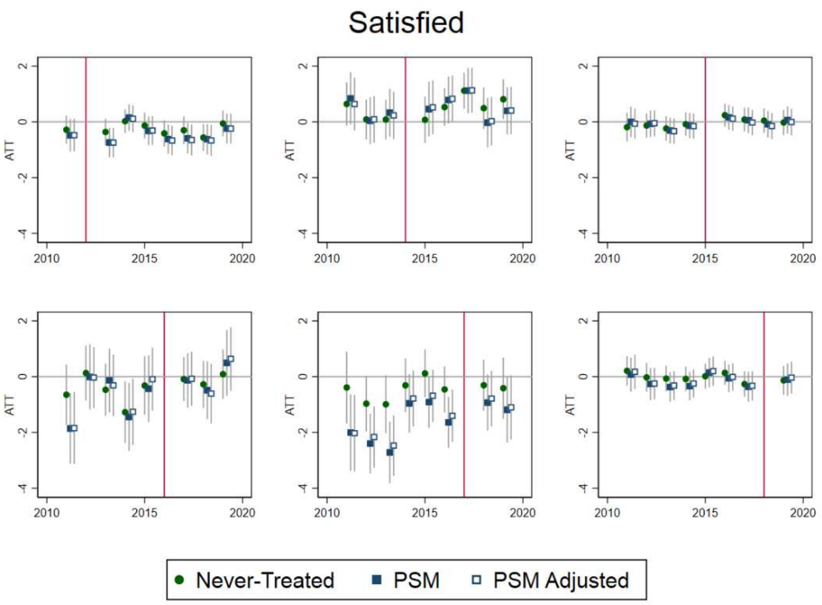

Supplementary Figure 5 Satisfied. Average treatment effect on the treated (ATT) for individual-level impacts of selective licencing (SL) on self-reported satisfied score among private renters in Greater London by year of SL introduction, 2011-2019. Time-varying covariates in PSM Adjusted were: age group, sex, native birth, and occupational class. Abbreviations: Propensity Score Matching (PSM).

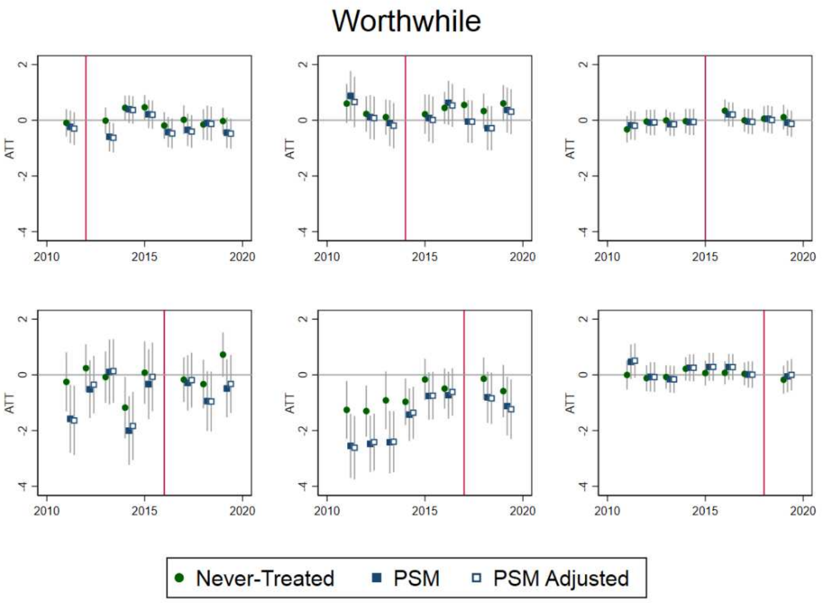

Supplementary Figure 6 Worthwhile. Average treatment effect on the treated (ATT) for individual-level impacts of selective licencing (SL) on self-reported worthwhile score among private renters in Greater London by year of SL introduction, 2011-2019. Time-varying covariates in PSM Adjusted were: age group, sex, native birth, and occupational class. Abbreviations: Propensity Score Matching (PSM).

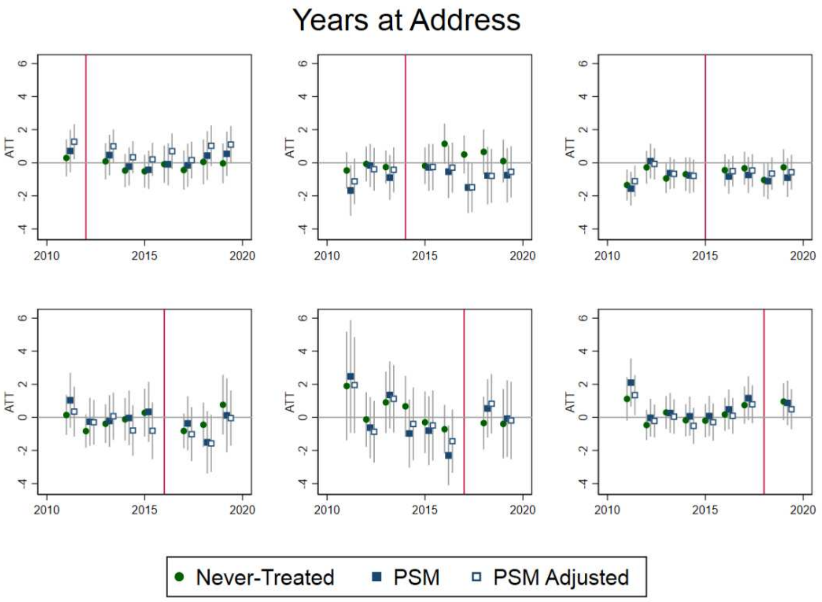

Supplementary Figure 7 Years at address. Average treatment effect on the treated (ATT) for individual-level impacts of selective licencing (SL) on self-reported years at address among private renters in Greater London by year of SL introduction, 2011-2019. Time-varying covariates in PSM Adjusted were: age group, sex, native birth, and occupational class. Abbreviations: Propensity Score Matching (PSM).

Supplementary Table 1 Baseline characteristics for Never-Treated and PSM control areas (LSOA) in Greater London, 2011. PSM controls were used for area-level impacts overall and for each year of treatment initiation, 2012 and 2014-2018, for individual-level impacts. Mean differences tested with a t-test except for Built pre-1945, which was tested with a Chi-square test (alpha=.05). Variables (Data source): Income deprived, Poor housing condition, No central heating, Unaffordable housing (Department for Communities and Local Government 2015b); Built pre-1945 (ONS 2021b); All other (ONS 2015a). Abbreviations: Lower Layer Super output Area (LSOA), Propensity Score Matched (PSM).

**Characteristics**	**All interventions**				
	**Treated**	**Never-Treated**		**PSM**	
	N=921	N=3,582	P-value	N=2,763	P-value
Children <16yr per pop	22.2	19.3	<.001	22.2	**0.839**
Adults 16-59yr per pop	64.1	64.3	**0.436**	63.8	**0.217**
Income deprived per pop	19.3	15.6	<.001	19.2	**0.823**
native birth per pop	60	65.3	<.001	60.6	**0.267**
Private rented%	25.9	23.4	<.001	25.3	**0.288**
Social rented%	22.7	22.5	**0.759**	22.8	**0.881**
Poor housing condition%	22.5	22.4	**0.621**	22.2	**0.298**
No central heating%	2.9	2.8	0.013	2.9	**0.906**
Overcrowded%	23.9	19.7	<.001	23.5	**0.402**
Unaffordable housing measure	2.4	1.7	<.001	2.4	**0.193**
Built pre-1945	-	-	<.001	-	**0.434**
**Characteristics**	**Interventions initiated in 2012**				
	**Treated**	**Never-Treated**		**PSM**	
	N=155	N=3,582	P-value	N=465	P-value
Children <16yr per pop	22.4	19.3	<.001	23.2	**0.153**
Adults 16-59yr per pop	68.2	64.3	<.001	67.7	**0.388**
Income deprived per pop	21.6	15.6	<.001	22.9	**0.15**
native birth per pop	46.5	65.3	<.001	47.4	**0.286**
Private rented%	33.9	23.4	<.001	33.4	**0.66**
Social rented%	28.9	22.5	<.001	31.9	**0.137**

Petersen J, *et al. BMJ Open* 2022; 12:e065747. doi: 10.1136/bmjopen-2022-065747

Poor housing condition%	22.8	22.4	**0.368**	22.7	**0.763**
No central heating%	2.8	2.8	**0.807**	2.8	**0.906**
Overcrowded%	34.9	19.7	<.001	34.8	**0.946**
Unaffordable housing measure	2.8	1.7	<.001	2.9	**0.259**
Built pre-1945	-	-	**0.122**	-	**0.186**
**Characteristics**	**Interventions initiated in 2014**				
	**Treated**	**Never-Treated**		**PSM**	
	N=110	N=3,582	P-value	N=330	P-value
Children <16yr per pop	25.9	19.3	<.001	26.1	**0.753**
Adults 16-59yr per pop	59.9	64.3	<.001	59.9	**0.871**
Income deprived per pop	24.2	15.6	<.001	25.2	**0.354**
native birth per pop	69.5	65.3	0.003	69.2	**0.83**
Private rented%	17.5	23.4	<.001	16.7	**0.361**
Social rented%	33	22.5	<.001	34.8	**0.44**
Poor housing condition%	23.1	22.4	**0.221**	22.3	**0.115**
No central heating%	3	2.8	**0.126**	2.9	**0.49**
Overcrowded%	19.9	19.7	**0.9**	20	**0.86**
Unaffordable housing measure	2.7	1.7	<.001	2.74	**0.793**
Built pre-1945	-	-	**0.498**	-	**0.715**
**Characteristics**	**Interventions initiated in 2015**				
	**Treated**	**Never-Treated**		**PSM**	
	N=394	N=3,582	P-value	N=1,182	P-value
Children <16yr per pop	21.5	19.3	<.001	21.3	**0.468**
Adults 16-59yr per pop	62.8	64.3	<.001	62.2	**0.083**
Income deprived per pop	17.5	15.6	<.001	16.6	**0.073**
native birth per pop	65.6	65.3	**0.664**	67	**0.123**
Private rented%	22.6	23.4	**0.238**	21.3	**0.084**
Social rented%	19.7	22.5	0.008	18.4	**0.196**
Poor housing condition%	22	22.4	**0.25**	21.4	**0.079**
No central heating%	3	2.8	0.005	2.9	**0.255**
Overcrowded%	19.5	19.7	**0.7**	18.4	**0.11**
Unaffordable housing measure	2.2	1.7	<.001	2	**0.063**
Built pre-1945	-	-	0.001	-	**0.723**
**Characteristics**	**Interventions initiated in 2016**				
	**Treated**	**Never-Treated**		**PSM**	
	N=28	N=3,582	P-value	N=84	P-value
Children <16yr per pop	17.8	19.3	**0.106**	16.5	**0.319**
Adults 16-59yr per pop	72.4	64.3	<.001	74	**0.42**
Income deprived per pop	23.5	15.6	<.001	22	**0.443**
native birth per pop	53.6	65.3	<.001	52	**0.413**
Private rented%	33.5	23.4	<.001	36.6	**0.341**
Social rented%	33.3	22.5	0.005	28.8	**0.239**
Poor housing condition%	25.8	22.4	0.003	28	**0.227**
No central heating%	3.1	2.8	**0.226**	3.8	**0.184**
Overcrowded%	35.3	19.7	<.001	36.3	**0.705**
Unaffordable housing measure	3.2	1.7	<.001	2.8	**0.21**
Built pre-1945	-	-	0.024	-	**0.827**
**Characteristics**	**Interventions initiated in 2017**				
	**Treated**	**Never-Treated**		**PSM**	
	N=59	N=3,582	P-value	N=177	P-value
Children <16yr per pop	21.9	19.3	<.001	21.6	**0.79**
Adults 16-59yr per pop	65.5	64.3	**0.219**	66	**0.618**
Income deprived per pop	20.3	15.6	<.001	21.3	**0.371**
native birth per pop	45.4	65.3	<.001	45.5	**0.961**
Private rented%	33.2	23.4	<.001	35.1	**0.345**
Social rented%	18.6	22.5	**0.141**	20.1	**0.547**

Petersen J, *et al. BMJ Open* 2022; 12:e065747. doi: 10.1136/bmjopen-2022-065747

Poor housing condition%	24	22.4	0.048	25.6	**0.148**
No central heating%	2.7	2.8	**0.564**	2.8	**0.701**
Overcrowded%	30.9	19.7	<.001	32.1	**0.407**
Unaffordable housing measure	3.5	1.7	<.001	3.3	**0.245**
Built pre-1945	-	-	0.027	-	**0.404**
**Characteristics**	**Interventions initiated in 2018**				
	**Treated**	**Never-Treated**		**PSM**	
	N=175	N=3,582	P-value	N=525	P-value
Children <16yr per pop	21.9	19.3	<.001	22.1	**0.806**
Adults 16-59yr per pop	64.3	64.3	**0.926**	64	**0.703**
Income deprived per pop	17.2	15.6	0.027	17	**0.787**
native birth per pop	58.8	65.3	<.001	58.8	**0.998**
Private rented%	27.8	23.4	<.001	27.5	**0.833**
Social rented%	17.2	22.5	<.001	17	**0.884**
Poor housing condition%	21.8	22.4	**0.266**	21.7	**0.708**
No central heating%	2.8	2.8	**0.866**	2.8	**0.747**
Overcrowded%	22.3	19.7	0.004	22	**0.785**
Unaffordable housing measure	2.2	1.7	<.001	2.2	**0.865**
Built pre-1945	-	-	0.001	-	**0.955**
