# Supplementary material for: Impact of selective licensing schemes for private rental housing on mental health and social outcomes in Greater London, England: a natural experiment study
Source: BMJ Open. 2022 Dec 23;12(12):e065747. doi: 10.1136/bmjopen-2022-065747 (PMC9791445; doi:10.1136/bmjopen-2022-065747)
Supplement: Author's manuscript [file bmjopen-2022-065747.draft_revisions.pdf]

BMJ Open is committed to open peer review. As part of this commitment we make the peer review history of every article we publish publicly available.

When an article is published we post the peer reviewers' comments and the authors' responses online. We also post the versions of the paper that were used during peer review. These are the versions that the peer review comments apply to.

The versions of the paper that follow are the versions that were submitted during the peer review process. They are not the versions of record or the final published versions. They should not be cited or distributed as the published version of this manuscript.

BMJ Open is an open access journal and the full, final, typeset and author-corrected version of record of the manuscript is available on our site with no access controls, subscription charges or pay-per-view fees (http://bmjopen.bmj.com).

If you have any questions on BMJ Open's open peer review process please email info.bmjopen@bmj.com

Impact of selective licensing schemes for private rental housing on mental health and social outcomes in Greater London, UK: a natural experiment study

Journal:	*BMJ Open*
Manuscript ID	bmjopen-2022-065747
Article Type:	Original research
Date Submitted by the Author:	16-Jun-2022

Complete List of Authors:	PETERSEN, JAKOB; London School of Hygiene and Tropical Medicine, ; UCL, Geography<br>Alexiou, Alexandros; University of Liverpool Faculty of Health and Life Sciences, Department of Public Health and Policy<br>Brewerton, David; Patient and public involvement representative<br>Cornelsen, Laura; London School of Hygiene and Tropical Medicine,<br>Courtin, Emilie; LSHTM,<br>Cummins, Steven; London School of Hygiene and Tropical Medicine, Public Health, Environments & Society<br>Marks, Dalya; London School of Hygiene & Tropical Medicine, Department of Social and Environmental Health Research<br>Seguin, Maureen ; London School of Hygiene and Tropical Medicine,<br>Stewart, Jill; Middlesex University, School of Science & Technology, Department of Natural Sciences<br>Thompson, Kevin; London Borough of Hackney, Head of Private Sector Housing<br>Egan, Matt; LSHTM, SEHR

Keywords:	EPIDEMIOLOGY, MENTAL HEALTH, PUBLIC HEALTH

BMJ

I, the Submitting Author has the right to grant and does grant on behalf of all authors of the Work (as defined in the below author licence), an exclusive licence and/or a non-exclusive licence for contributions from authors who are: i) UK Crown employees; ii) where BMJ has agreed a CC-BY licence shall apply, and/or iii) in accordance with the terms applicable for US Federal Government officers or employees acting as part of their official duties; on a worldwide, perpetual, irrevocable, royalty-free basis to BMJ Publishing Group Ltd ("BMJ") its licensees and where the relevant Journal is co-owned by BMJ to the co-owners of the Journal, to publish the Work in this journal and any other BMJ products and to exploit all rights, as set out in our licence.

The Submitting Author accepts and understands that any supply made under these terms is made by BMJ to the Submitting Author unless you are acting as an employee on behalf of your employer or a postgraduate student of an affiliated institution which is paying any applicable article publishing charge ("APC") for Open Access articles. Where the Submitting Author wishes to make the Work available on an Open Access basis (and intends to pay the relevant APC), the terms of reuse of such Open Access shall be governed by a Creative Commons licence – details of these licences and which Creative Commons licence will apply to this Work are set out in our licence referred to above.

Other than as permitted in any relevant BMJ Author's Self Archiving Policies, I confirm this Work has not been accepted for publication elsewhere, is not being considered for publication elsewhere and does not duplicate material already published. I confirm all authors consent to publication of this Work and authorise the granting of this licence.

For peer review only

Title

Impact of selective licensing schemes for private rental housing on mental health and social outcomes in Greater London, UK: a natural experiment study

Authors

Authors [Alphabetical order except first/last]
Jakob Petersen[1]*, PhD, research fellow
Alexiou, Alexandros[2], PhD, research associate
David Brewerton, Patient and Public Involvement representative
Laura Cornelsen[1], PhD, associate professor
Emilie Courtin[1], PhD, assistant professor
Steven Cummins[1], PhD, professor
Dalya Marks[1], PhD, associate professor
Maureen Seguin[1], PhD, research fellow
Jill Stewart[3], PhD, senior lecturer
Kevin Thompson[4], BSc, head of private housing
Matt Egan[1], PhD, professor

Affiliations

1. London School of Hygiene & Tropical Medicine, Public Health, Environments and Society, 15-17 Tavistock Place, London, WC1H 9SH, UK.
2. University of Liverpool, Department of Public Health, Policy and Systems, Waterhouse Building, Block B, Brownlow Street, Liverpool, L69 3GF, UK.
3. Middlesex University, Natural Sciences, The Burroughs, Hendon, London NW4 4BT, UK.
4. London Borough of Hackney, 1 Hillman Street, London, E8 1DY, UK.

*) Corresponding author (JP): jakob.petersen1@lshtm.ac.uk; https://orcid.org/0000-0002-6659-7028

For peer review only

Abstract (269/300 words)

Objectives

To assess primary impact of Selective Licencing (SL), an area-based intervention in the private rented housing market, on individual self-reported anxiety and neighbourhood mental health (MHI - mental healthcare index) and secondary impacts on antisocial behaviour (ASB), population turnover, and self-reported wellbeing.

Design

Difference-in-differences (DiD) was used to evaluate effects of SL schemes initiated 2012-2018. 921 intervention areas (Lower Super Output Areas) were matched 3:1 using propensity scores derived from sociodemographic and housing variables (N=3.684 incl. controls). Average Treatment effect on Treated (ATT) was calculated for multiple time period DiD in area-level analyses. Canonical DiD was used for individual-level analysis by year of treatment initiation while adjusting for age, sex, native birth, and occupational class.

Setting

Intervention neighbourhoods and control areas in Greater London, UK, 2011-2019.

Participants

We sampled 4,474 respondents renting privately in intervention areas (N=17,347 incl. controls) in Annual Population Survey and obtained area-level MHI population data.

Interventions

Private landlords in SL areas must obtain a licence from the local authority, allow inspection, and maintain minimum housing standards.

Results

ATT after 5 years was significantly lower for MHI (-7.5%, 95% confidence intervals -5.6;-8.8) than controls. Antidepressant treatment days per population reduced by -5.4% (-3.7;-7.3), mental health benefit receipt by -9.6% (-14;-5.5) and proportion with depression by -12% (-7.7;-16.3). ASB reduced by -15% (-21;-8.2). Population turnover increased by 26.5% (22.1;30.8). Sensitivity analysis suggests overlap with effects of London 2012 Olympic regeneration. No clear patterns were observed for self-reported anxiety.

Conclusions

We found associations between SL and reductions in area-based mental healthcare outcomes and ASB, while population turnover increased. A national evaluation of SL is feasible and necessary.

Article Summary

Strengths and limitations of this study

- This is the first-ever evaluation of mental health and social outcomes of selective licencing schemes.
- The multiple time period DiD design assesses impacts of the staggered area-based intervention over and above a host of other factors that influence mental health and wellbeing.
- A limitation is that it is inherently not possible to eliminate selection bias due to non-random treatment allocation of selective licencing schemes.
- As a limitation, the area-level findings of this study could not be complemented by individual-level data due to data sparsity in the survey sample.

Key words

Social determinants of health; Housing; Public health intervention; Evaluation; Natural experiment

For peer review only

For peer review only - http://bmjopen.bmj.com/site/about/guidelines.xhtml

Introduction

Housing quality affects health [1]. Poor quality homes present numerous environmental risks to residents' health, including risks of injury, physical illnesses linked to cold, damp, and indoor pollution, and risks to mental health and wellbeing [2]. The costs to the English healthcare system attributed to poor housing rivals those associated with hazards such as smoking and alcohol consumption [3,4]; costing an estimated £1.4bn in 2021 [4]. The unequal distribution of poor-quality homes across the population correlates with other social inequalities in health [5].

Housing improvement interventions can have a positive impact on residents' health, including mental health and wellbeing, particularly when targeted at those most in need [2,6–9]. Therefore, strategies for improving population health and health equity often include housing improvement [1,10].

Housing quality improved between 2000 and 2019 in England across all sectors, but conditions are consistently worse in the private rented sector (PRS) compared to owner-occupied and the social rented sector [11]. For instance, the proportion of homes failing to meet the criteria of the Decent Homes Standard in 2019 was 23% in PRS compared to 12% in the social rented sector and 16% for owner occupied homes. The PRS doubled between 2000 and 2019 in tandem with falling affordability of private homes and shrinking of the social housing sector [11].

The need for action to improve PRS quality has been recognised by UK governmental bodies such as the National Audit Office [12] as well as the Chartered Institute for Environmental Health [13]. In 2006, local authorities gained discretionary powers to regulate privately rented homes through 'selective licencing' (SL) schemes under Housing Act 2004 [14]. In SL schemes, landlords in areas targeted by local authorities must pay for a licence, allow inspection, and carry out work necessary to maintain minimum housing standards. Fees are typically around £600 for a 5-year license. SL schemes can only be implemented following a consultation with local stakeholders and only some local authorities have implemented SL to date.

There are very few experimental and long-running studies of the links between housing and health due to lack of acceptability, ethics, treatment blinding, and funding [2,9]. The evidence therefore mainly comes from observational, and often short-term, studies of both individuals and neighbourhoods [7,9]. Although housing improvement interventions have on occasion been implemented as part of a randomised controlled study [9], they are more typically implemented in ways that would require natural experimental impact evaluations.

A systematic review of the effect of housing improvement on health outcomes published in 2013 found the clearest evidence for interventions around thermal comfort, especially if targeted at people with the highest needs (poorer baseline health and/or socio-economic status) [9]. Being able to heat the home economically had positive impacts on health outcomes (general health, mental health, respiratory health, reduced absences from work and school) as well as facilitating better use of indoor space for the residents. In 2019, a systematic review of English-language studies from high-income countries found, in addition to heating, health benefits from improved ventilation, improved water supply, and removal of indoor hazards [2]. Another recent review found evidence that mental health, wellbeing and other outcomes are at risk in the PRS, although the evidence base for interventions that might improve the sector was poor [15].

Government guidance on SL states that schemes can be implemented to combat area-level problems such as deprivation or ASB [16]. Antisocial behaviour (ASB) is defined in the law as behaviours causing 'harassment, alarm, or distress', which ranges from littering to complaints over rowdy neighbours [17]. Although housing improvement interventions can lead to neighbourhood-level improvements [7,9], the mechanisms by which SL may achieve such impacts (incl. on ASB) is not understood. We hypothesise that improved property and positive feelings towards an area may link to reduced ASB. However, unintended impacts of SL, including potential harms, can also be hypothesised. For example, it is possible that costs for license fees and required improvements are passed on to tenants, and leads to evictions. As a result, households experiencing hardships may be displaced to other localities or face homelessness.

For peer review only - http://bmjopen.bmj.com/site/about/guidelines.xhtml

There have not been any systematic attempts to measure the potential impact of SL on mental health, wellbeing, and ASB. This natural experiment study addresses this gap and functions as a feasibility study for a national evaluation of the impacts of SL. This paper primarily evaluates impacts on individual self-reported anxiety and neighbourhood mental healthcare in areas that have implemented SL compared to controls in Greater London. Secondarily, it evaluates self-reported wellbeing outcomes at the individual level, and ASB and population turnover at the area level.

Materials and methods

A protocol paper describing the methodology in more detail has been previously published [18].

Patient and public involvement

We consulted two Patient and Public Involvement representatives throughout the project.

Interventions

We obtained details of the spatial and temporal extent of all current and historic SL schemes through Freedom of Information requests (FOI) to all 33 local authorities in Greater London from when first enacted in 2006 to the end of 2019. We included all schemes initiated in or before 2018 in the analyses (Table 1). To standardise the area-based data for analysis, conversion weights were calculated based on the number of 2011 Census enumeration postcodes [19] falling into small intercepts between the de facto geographical unit and the unit of analysis, Lower Layer Super Output Areas 2011 (LSOA; approx. 1,700 average population) [20]. LSOA units that were only partially under treatment (conversion weights >0 and <1) were removed from both the treatment and control pool prior to analysis (N=17 LSOA excluded). Data from two boroughs that introduced street-level schemes (N=279 LSOA excluded), i.e. Hammersmith & Fulham and Southwark, and a single electoral ward that was used as a pilot in Newham (N=9 LSOA excluded) were also excluded.

Table 1 Selective Licencing (SL) schemes in Greater London up until 2018 [Year/Local authority]. Geographies were standardised to fully treated LSOA units. Population estimates are based on Census 2011. APS Private renter interviews in 2011-2019 tabulated by year of treatment initiation. Abbreviations: Annual Population Survey (APS), Lower Layer Super Output Area (LSOA).

**Scheme**	**LSOA spatial units** N	**Population 2011** N	**Treated private renters** Annual Mean (Min;Max)	Total N	**Control private renters** Annual Mean (Min;Max)	Total N	**Treated + Controls** Total N
2012 Newham	155	291,351	110 (61;143)	994	298 (175;393)	2,686	3,680
2014 Barking-Dagenham	110	185,911	66 (54;73)	590	104 (83;132)	937	1,527
2015 Brent	23	47,476					
2015 Waltham Forest	144	258,249					
2015 Croydon	220	363,378					
2015 Harrow	7	11,653	156 (116;202)	1,406	549 (428;628)	4,938	6,344
2016 Harrow	6	11,394					
2016 Tower Hamlets	22	38,354	22 (16;35)	200	57 (25;82)	511	711
2017 Ealing	43	77,024					
2017 Redbridge	16	28,789	31 (11;50)	278	135 (74;181)	1,214	1,492
2018 Harrow	14	24,491					
2018 Brent	42	75,793					
2018 Bexley	13	23,499					
2018 Hackney	15	26,366					
2018 Redbridge	91	164,845	112 (74;141)	1,006	287 (225;337)	2,587	3,593
Total	921	1,628,573	-	4,474	-	12,873	17,347

Outcomes – Area-level impacts

Small Area Mental Health Index (SAMHI) scores were obtained by year and small area (LSOA) [21]. SAMHI combines data on mental healthcare from multiple sources into a single index, i.e. National

For peer review only - http://bmjopen.bmj.com/site/about/guidelines.xhtml

Health Service (NHS) data on z-score standardised mental health-related admission (referred to as ADMISSION, hereinafter), antidepressant treatment days per population (PRESCRIPTION), primary care data on the percentage of the population diagnosed with depression (DIAGNOSIS), and Department for Work and Pensions data on the percentage of population in receipt of mental health-related benefits (BENEFITS). The SAMHI score is proportional to the overall burden on the healthcare system, i.e. an increase signifies a worsening outcome. Each of the underlying SAMHI indicators (ADMISSION, PRESCRIPTION, DIAGNOSIS, BENEFITS) were, according to protocol, studied individually if a positive result was obtained with SAMHI itself.

High levels of ASB is the most common reason for local authorities implementing SL [14], so we assessed the incidence of police-recorded ASB by year and LSOA as a secondary outcome [22]. Data from a population turnover index were studied as a secondary outcome to test an association between SL exposure and moves [23].

The population turnover index data are estimates based on a combination of electoral roll and consumer data (CDRC Residential Mobility Index 2020) [23]. The index is released as a cumulative and the annual proportion of households that will move in the coming year was derived for these analyses. The background for the index is the absence of officially released data other than the decennial censuses. The starting point for the index is the edited electoral roll (i.e. the publicly available version without data on individuals who have opted out for privacy reasons and to avoid direct marketing) complemented with data on names and addresses of consumers collected by commercial data services companies [23].

Statistical methods – Area-level impacts

A Difference-in-Differences (DiD) approach was deployed for the area-level impacts with three different strategies for controls: 1. All never-treated areas, 2. Propensity Score Matched control (PSM) areas (the primary control strategy), and 3. Not-yet-treated areas. The PSM controls were intended as a counterfactual based on measured baseline area characteristics, while the Not-yet-treated controls, a counterfactual for unmeasured characteristics. Local authorities can justify the introduction of SL based on locally held data, e.g. poor housing conditions. This is what we mean by the term unmeasured characteristics in these analyses. Never-treated controls were studied as a check of bias potentially introduced by the matching and trimming of the sample in PSM. The PSM used as far as possible pre-intervention sociodemographic, housing, and neighbourhood characteristics from the 2011 Census, Indices of Multiple Deprivation, and official dwelling age data (Supplementary Table 1) [24–26]. The matching was carried out with the Stata module KMATCH [27]. The parallel trend assumption was checked visually in the DiD plots.

Homeowners and social renters were by design studied in parallel with private renters for falsifiability checks. SL should only directly affect private renters and any effects detected for private renters could therefore also be challenged by studying not directly affected groups in the same intervention areas. Given the staggered nature of the intervention, a DiD method for comparing multiple time periods were used [28]. The number of intervention LSOA units was 921 and the total number of LSOA in the DiD-PSM analysis was 3,684 (incl. 3 controls per 1 intervention area) (Table 1). The average treatment effect on the treated (ATT) estimated by the DiD was given as ATT% for Ln-transformed indicators (BENEFITS, ADMISSION, ASB), ATT% = -100*(1-exp(ATT)). ATT% was for comparison also calculated for untransformed variables relative to the baseline value.

Outcomes – Individual-level impacts

Data on adult respondents in Annual Population Survey (APS) in England, 2011-2019, were obtained from Office for National Statistics (ONS) [29]. Among these, we identified 4,474 private renters exposed to the intervention (total number of renters incl. controls, N=17,347) (Table 1). The four subjective health and wellbeing questions in APS (aka. ONS4) with scores from 0 to 10 were assessed. The anxiety question was the primary outcome and the other questions on subjective wellbeing (happiness, life satisfaction, whether the things you do in life are worthwhile), secondary outcomes. Data on how long the respondent had lived at the address (asked in categories and recoded

For peer review only - http://bmjopen.bmj.com/site/about/guidelines.xhtml

to mid-category values for these analyses) were studied at the same time as a proxy of residential stability.

Statistical methods – Individual-level impacts

A canonical DiD approach was deployed for the individual-level impacts by year of treatment initiation in 2012, 2014, and 2015, respectively [30]. Schemes introduced the same year were pooled for statistical efficiency. Three different controls were used: 1. Never-treated, 2. PSM controls, and 3. PSM adjusted for age, sex, native birth, and occupational class [31].

Results

The size of the different SL schemes in terms of fully treated LSOA units, population, and number of private renters captured in the APS data can found in Table 1.

The overall trend in the composite mental healthcare indicator, SAMHI, was a gradual increase in burden during 2011-2019, while antisocial behaviour calls declined sharply in 2011-2015 and then more slowly for most control and treatment groups (Figure 1). Population turnover fluctuated during the study period. The trends for the underlying SAMHI indicators are shown in Supplementary Figure 1.

The trends for the APS outcomes showed a slight improvement with a decline in how anxious the respondent felt the day before the interview and a slight increase for the other subjective wellbeing indicators (happy, satisfied, worthwhile) and years at address. The trends for the different SL schemes by year of treatment initiation were similar yet noisier presumably due to small number issues in the APS sample (Figure 2).

The ATT with PSM controls after 5 years of intervention were significantly different from baseline for all area-based outcomes, SAMHI, antisocial behaviour calls, and population turnover (Table 2, Figure 3). Further analysis of the underlying SAMHI indicators showed similar positive results for antidepressant prescribing, depression diagnosis, and mental health-related benefits, while no clear patterns were seen with mental health-related hospital admission (Supplementary Figure 2). The average number of antidepressant treatment days per population in treatment areas at baseline was 13.1. This number reduced by -0.71 days (95% confidence intervals, -0.95 to -0.48) after 5 years of intervention (Table 2), i.e. a -5.4% (-3.7;-7.3) reduction from the baseline in relative terms. Mental health-related benefits were received by 2.4% of the population at baseline and reduced by -9.6% (-14 to -5.5), i.e. -0.23 (-0.13;-0.34) percentage point change in absolute terms. The proportion of the population diagnosed with depression was 3.5% at baseline and reduced by -0.42 percentage points (-0.57 to -0.27), i.e. -12% (-7.7;-16.3) reduction of baseline in relative terms. Antisocial behaviour calls per 10,000 population was 537 at baseline and reduced (i.e. improved) by -15% (-21 to -8.2). Population turnover, as in the proportion of household that will move in the coming year, was 5.2% at baseline and increased by 1.38 percentage points, i.e. 26.5% (22.1;30.8) in relative terms.

A sensitivity check of excluding the sole scheme initiated in 2012 was carried out. Apart from being the earliest London scheme, it also concerned the borough that was centre for the 2012 London Olympics (we here term it the 'Olympic' scheme). The results showed no 5-year results with SAMHI, similar reduction in antisocial behaviour calls, and a more modest increase in population turnover (Supplementary Figure 3).

There were no clear patterns from the individual level analyses of APS data (Figure 4, Supplementary Figures 4-7).

For peer review only - http://bmjopen.bmj.com/site/about/guidelines.xhtml

Table 2 Average Treatment effect on Treated (ATT) for area and individual impacts after 3, 5, and 7 years with PSM controls. ATT given as ATT% for Ln-transformed indicators (Benefits, Admission, ASB). For individual impacts, ATT adjusted for time-varying sociodemographic covariates and relate to the interventions initiated in 2012, 2014, and 2015. ATT values significant at 5% alpha level shown in bold face. Abbreviations: Antisocial Behaviour calls (ASB), Not Applicable (N/A), Propensity Score Matching (PSM).

**Indicator**	**Unit**	**Baseline mean (2011)**			**ATT**		**ATT%**
		**Never-Treated**	**Treated**	**3-year**	**5-year**	**7-year**	**5-year**
Area impacts – Interventions initiated 2012-2018							
SAMHI	Index score	-1.4	-1.6	**-.03 (-.05;-.02)**	**-.12 (-.14;-.09)**	**-.27 (-.29;-.24)**	**-7.5% (-5.6;-8.8)**
-Prescription	Antidepressant treatment	15.5	13.1	**-.19 (-.33;-.04)**	**-.71 (-.95;-.48)**	**-1.81 (-2.13;-1.49)**	**-5.4% (-3.7;-7.3)**
-Benefits	%pop	2.5	2.4	**-8.5% (-11;-5.8)**	**-9.6% (-14;-5.5)**	-4.3% (-10;2.3)	**-9.6% (-14;-5.5)**
-Diagnosis	%pop	4.3	3.5	**-.17 (-.26;-.08)**	**-.42 (-.57;-.27)**	**-1.5 (-1.62;-1.37)**	**-12% (-7.7;-16.3)**
-Admission	z-score	-.71	-.54	24% (-11;72)	**-44% (-66;-9.9)**	-23% (-58;40)	**-44% (-66;-9.9)**
ASB	Calls per 10k pop	495	537	-3.8% (-7.8;.41)	**-15% (-21;-8.2)**	**-12% (-22;-1.2)**	**-15% (-21;-8.2)**
Pop turnover	%households moving	5.5	5.2	**0.39 (.29;.5)**	**1.38 (1.15;1.6)**	**0.86 (0.57;1.14)**	**26.5% (22.1;30.8)**
Individual impacts – Interventions initiated 2012							
Anxious	0-10 scale	3.5	4.2	0.09 (-.72;.9)	0.35 (-.5;1.21)	0.59 (-.28;1.45)	8.3% (-11.9;5)
Happy	0-10 scale	7.2	7.1	-.14 (-.78;.5)	**-.68 (-1.36;-.005)**	0.03 (-.62;.68)	**-9.6% (-19.2;-0.1)**
Satisfied	0-10 scale	7.2	7.2	-.31 (-.83;.2)	**-.65 (-1.2;-.01)**	-.24 (-.78;.29)	**-9% (-16.7;-0.1)**
Worthwhile	0-10 scale	7.5	7.4	0.2 (-.3;.7)	-.4 (-.99;.18)	-.48 (-1.02;.06)	-5.4% (-13.4;2.4)
Years at address	Years	2.9	3.6	0.2 (-.79;1.12)	0.16 (-.95;1.27)	1.1 (-.03;2.23)	4.4% (-26.4;35.3)
Individual impacts – Intervention initiated 2014							
Anxious	0-10 scale	3.5	3.2	-1.05 (-2.35;.26)	-.001 (-1.29;1.28)	N/A	0% (-40.3;40)
Happy	0-10 scale	7.2	7.2	0.02 (-.99;1.02)	-.41 (-1.44;.63)	N/A	-5.7% (-20;8.8)
Satisfied	0-10 scale	7.2	7.1	**1.13 (.32;1.93)**	0.4 (-.44;1.25)	N/A	5.6% (-6.2;17.6)
Worthwhile	0-10 scale	7.5	7.6	-.05 (-.82;.71)	0.31 (-.5;1.11)	N/A	4.1% (-6.6;14.6)
Years at address	Years	2.9	2.7	-1.48 (-2.99;.03)	-.55 (-2.01;1)	N/A	-20.4% (-74.4;37)
Individual impacts – Intervention initiated 2015							
Anxious	0-10 scale	3.5	3.3	0.71 (-.03;1.44)	N/A	N/A	N/A
Happy	0-10 scale	7.2	7.5	-.19 (-.73;.35)	N/A	N/A	N/A
Satisfied	0-10 scale	7.2	7	-.15 (-.62;.31)	N/A	N/A	N/A
Worthwhile	0-10 scale	7.5	7	0.01 (-.48;.5)	N/A	N/A	N/A
Years at address	Years	2.9	2.7	-.65 (-1.64;.33)	N/A	N/A	N/A

For peer review only

Discussion

The study found improvements in area-based mental health outcomes and antisocial behaviour calls (ASB), while population turnover increased. Conversely, the results for self-reported anxiety and other individual-level indicators were inconclusive due to the small sample size of the APS data.

The results indicate potential benefits of SL schemes beyond their 5-year cycle, especially for reduction of ABS. We cannot exclude that at least part of the change could be due to gentrification and we saw an increase in population turnover to suggest this. Future quantitative studies of area-based impacts should therefore assess whether gentrification effects can be ruled out.

These first findings may be confounded by the fact that the earliest scheme overlapped with urban regeneration projects in connection with the 2012 London Olympics. A sensitivity check excluding the 'Olympic' scheme did not show any reduction in the main area-based mental healthcare indicator, SAMHI. There was however a similar reduction in ASB and a more modest increase in population turnover after five years (both statistically significant). Studies of the impacts of the Olympic event itself and its legacy have notably been mixed. A telephone survey of residents in London, Berlin, and Paris in 2011-2013 found a short-lived increase in subjective wellbeing for Londoners during the event [32]. A longitudinal cohort study of adolescents and their families living close to the Olympic site compared to those living further away found no changes in self-reported health behaviours or health outcomes (including subjective wellbeing) from before to 18 months after the event [33]. Co-occurring policies are a potential threat to the validity of our estimates [34]. Future research should therefore repeat our analysis when longer time series are available and more schemes can be studied in London and nationally to disentangle the effects of SL from the long-term effects of the urban regeneration such as those surrounding the London Olympics.

In this study, we defined mental health broadly with indicators ranging from self-reported wellbeing to mental health hospital admission. It is clear that the social surveys that cover subjective wellbeing are typically not designed for sub-regional analysis. Administrative or routinely collected data are, on the other hand, more scalable, yet only capture the more extreme end of the mental health scale, and often very hard to access for researchers due to information governance strictures. Recent developments triggered by the COVID19 pandemic however have opened up new opportunities for secure data linkage at patient address level [35]. This development is promising for the evaluation of housing policies such as SL.

A 10-year natural experiment study of healthcare service use in social housing residents age 60+ years in the UK found that those who received improvements to their kitchens, bathrooms or front doors, among other kinds of improvement, presented less often with common mental health disorders than those who did not receive these improvements [8]. A 5-year study (GoWell) of the impact of housing improvements on self-reported mental health and wellbeing among social housing residents found additional positive effects of renewing fabric works, i.e. carpets, curtains, and blinds [6]. The GoWell study also found a positive correlation between self-reported mental health and wellbeing among social housing residents and urban regeneration spending, which locally could cover internal housing, external housing, neighbourhoods, as well as community project investments. It was the residents with the highest needs, who resided in the worst housing in the most rundown neighbourhoods, receiving the highest urban regeneration investment, who ultimately showed the greatest improvements in self-reported mental health and wellbeing [7]. Another UK natural experiment study of urban regeneration found positive effects for residents' mental health [36]. These studies support the link between housing improvement and mental health and wellbeing suggested by the present study.

A recent systematic review on housing and health reported randomised controlled trial evidence about mental health benefits for both children and adults in relation to improvements of heating and

For peer review only - http://bmjopen.bmj.com/site/about/guidelines.xhtml

ventilation [2]. Another recent systematic review of earlier housing disadvantage and poor mental health outcomes reported clear correlations, but also called for more studies to elucidate mechanisms [37]. Another review identified PRS as a growing yet overlooked sector with wide-ranging needs including mental health needs [15]. The review also acknowledged a current lack of evidence about effective interventions. Taking together, the reviews highlight a need for more and better evidence of social polices aiming to improve housing quality including in PRS.

Although by definition a non-crime, reduction of ASB [17] is considered a key objective for the policing of London based on consultation and social surveys on the perception of crime [38]. It is common for local authorities to use reduction of ASB as a justification for SL [14], although the mechanisms for this are not stated [16]. Hypothetically, it could happen through gentrification or be linked to improved property, and positive feelings towards the neighbourhood. Interestingly, we found that ASB reduced after 4-5 years of SL – even when we excluded the 'Olympic' scheme. Further studies should examine the reasons for the ASB calls, e.g. whether the calls concern neighbours.

A strength of the study is our use of the DiD design, which assesses impacts over and above a host of other factors that influence mental health and wellbeing. In addition, the multiple time period comparison DiD summarises the effect of a staggered intervention such as SL in a single analysis [28]. This step also enables Not-yet treated as control of unmeasured factors associated with treatment allocation. Never-treated controls were included in true effects could be masked by overmatching in the PSM. Reassuringly, the different controls generally yielded similar results in this study.

The area-level findings should be backed up by individual-level findings specific to private renters and free of ecological bias [39]. In this case, we found that the APS sample data were too sparsely populated to create robust panel units over time and that many of the smaller schemes therefore could not be properly assessed. We instead deployed a canonical DiD approach and analysed SL by year of treatment initiation. The results were however inconclusive due the large variation associated with small sample size. Future studies should include data at the national level to reach higher numbers. At the same time, linkage between administrative housing and administrative healthcare data should be explored.

This study is to our knowledge the first to use SAMHI [21] and CDRC Residential Mobility index [23] in an evaluation of an area-based policy such as SL. There was much higher precision in the SAMHI sub-scores, PRESCRIPTION and DIAGNOSIS, than in BENEFITS and ADMISSION. The results with ADMISSION were particularly unprecise and variable. CDRC Residential Mobility index provides yearly estimates of moves, whereas the 'gold standard', the Census flow data, in contrast are only released every ten years [40]. The trend in annual proportion of households that will move in the coming year showed a great deal of fluctuation in itself. Due to the DiD design of this study, 'global' fluctuations are in themselves not prohibitive for an evaluation of an area-based intervention. Future releases should nonetheless examine whether the fluctuations can be explained.

The PSM used as far as possible pre-intervention sociodemographic and housing variables. It is possible that the matching could produce a more realistic counterfactual if more pre-intervention data relevant to treatment allocation and/or outcome risk factors become available in the future.

Conclusions

We found early indications of a reduction in area-based mental health outcomes and ASB, while population turnover increased. Results from the individual-level analysis of APS data were inconclusive; possibly due to sample size issues. Longer time series are needed to disentangle SL

For peer review only - http://bmjopen.bmj.com/site/about/guidelines.xhtml

from Olympic regeneration. Further studies specific to private renters and gentrification effects are needed. Overall, we argue that a national evaluation of SL is feasible and necessary.

Contributorship

All authors contributed to the conception, study design, data interpretation, and approved the submitted version. JP contributed to data acquisition and drafted the first manuscript.

Competing interests

The investigators declare no competing interests of financial nature and, as a whole, counts both homeowners and tenants.

Funding statement

This study is funded by the National Institute for Health Research (NIHR) School for Public Health Research (SPHR) (Grant Reference Number PD-SPH-2015). The views expressed are those of the authors and not necessarily those of the NIHR or the Department of Health and Social Care.

Data sharing

The data supporting the findings of this study were obtained under licence and as such not available to other researchers. The data are, however, available from Office for National Statistics subject to ethical and scientific approval. This work was produced using statistical data from ONS [Annual Population Survey]. The use of the ONS statistical data in this work does not imply the endorsement of the ONS in relation to the interpretation or analysis of the statistical data. This work uses research datasets which may not exactly reproduce National Statistics aggregates.

Ethics approval

Ethical approval was obtained from London School of Hygiene and Tropical Medicine's Ethics Committee (reference number 26481) and London Borough of Hackney.

References

1 WHO. WHO Housing and health guidelines. Geneva, Switzerland: 2018. https://www.who.int/publications-detail-redirect/9789241550376 (accessed 21 Jul 2021).

2 Ige J, Pilkington P, Orme J, *et al.* The relationship between buildings and health: a systematic review. *J Public Health* 2019;**41**:e121–32. doi:10.1093/pubmed/fdy138

3 Roys M, Nicol S, Garrett H, *et al. The full cost of poor housing*. Bracknell, UK: : BRE & IHS Global Ltd 2016.

4 Garrett H, Mackay M, Nicol S, *et al.* The cost of poor housing in England. Watford, UK: : BRE Group 2021.

5 Gibson M, Petticrew M, Bambra C, *et al.* Housing and health inequalities: a synthesis of systematic reviews of interventions aimed at different pathways linking housing and health. *Health Place* 2011;**17**:175–84. doi:10.1016/j.healthplace.2010.09.011

6 Curl A, Kearns A, Mason P, *et al.* Physical and mental health outcomes following housing improvements: evidence from the GoWell study. *J Epidemiol Community Health* 2015;**69**:12–9. doi:10.1136/jech-2014-204064

7 Egan M, Kearns A, Katikireddi SV, *et al.* Proportionate universalism in practice? A quasi-experimental study (GoWell) of a UK neighbourhood renewal programme's impact on health inequalities. *Soc Sci Med 1982* 2016;**152**:41–9. doi:10.1016/j.socscimed.2016.01.026

For peer review only - http://bmjopen.bmj.com/site/about/guidelines.xhtml

8 Rodgers SE, Bailey R, Johnson R, *et al.* Health impact, and economic value, of meeting housing quality standards: a retrospective longitudinal data linkage study. *Public Health Res* 2018;**6**:1–104. doi:10.3310/phr06080

9 Thomson H, Thomas S, Sellstrom E, *et al.* Housing improvements for health and associated socio-economic outcomes. *Cochrane Database Syst Rev* 2013;:CD008657. doi:10.1002/14651858.CD008657.pub2

10 Marmot M, Allen J, Boyce T, *et al.* Health Equity in England: The Marmot Review 10 Years On. Health Found. 2020.https://www.health.org.uk/publications/reports/the-marmot-review-10-years-on (accessed 10 Jul 2020).

11 Ministry of Housing, Communities and Local Government. English Housing Survey: headline report 2019-2020. London, UK: 2020. https://assets.publishing.service.gov.uk/government/uploads/system/uploads/attachment_data/file/945013/2019-20_EHS_Headline_Report.pdf (accessed 30 Mar 2021).

12 National Audit Office. Regulation of private renting. London, UK: : National Audit Office 2021.

13 Chartered Institute of Environmental Health. A National Registration Scheme for the private rented sector - Parliamentary Briefing 10 July 2019. London, UK: : Chartered Institute of Environmental Health 2019.

14 Lawrence S, Wilson P. An independent review of the use and effectiveness of selective licensing. London, UK: : Ministry of Housing, Communities and Local Government 2019. https://www.gov.uk/government/publications/selective-licensing-review (accessed 30 Mar 2021).

15 Harris J, McKee K. Health and wellbeing in the private rented sector Part 1: Literature review. Glasgow: : UK Collaborative Centre for Housing Evidence 2021. https://housingevidence.ac.uk/publications/health-and-wellbeing-in-the-private-rented-sector-part-1-literature-review/ (accessed 10 Nov 2021).

16 Department for Communities and Local Government. Selective licensing in the private rented sector: a guide for local authorities. London, UK: 2015. https://www.gov.uk/government/publications/selective-licensing-in-the-private-rented-sector-a-guide-for-local-authorities (accessed 30 Mar 2021).

17 HM Government. Anti-social Behaviour, Crime and Policing Act 2014. Statute Law Database 2014. https://www.legislation.gov.uk/ukpga/2014/12/contents (accessed 23 Jul 2021).

18 Petersen J, Seguin M, Alexiou A, *et al.* Assessing the impact of selective licencing schemes for private rental housing on mental health and well-being: protocol for a mixed-method natural experiment study in Greater London, UK. *BMJ Open* 2022;**12**:e057711. doi:10.1136/bmjopen-2021-057711

19 ONS. Postcode Headcounts and Household Estimates - 2011 Census. Nomis - Off. Labour Mark. Stat. 2021.https://www.nomisweb.co.uk/census/2011/postcode_headcounts_and_household_estimates (accessed 21 Jul 2021).

20 Simpson L, Yu A. Public access to conversion of data between geographies, with multiple look up tables derived from a postal directory. *Comput Environ Urban Syst* 2003;**27**:283–307. doi:10.1016/S0198-9715(02)00018-2

For peer review only - http://bmjopen.bmj.com/site/about/guidelines.xhtml

21 Daras K, Barr B. Small Area Mental Health Index (SAMHI) 2.0, Place-based Longitudinal Data Resource. 2020. https://pldr.org/dataset/2noyv/small-area-mental-health-index-samhi (accessed 2 Jul 2021).

22 Police.uk. data.police.uk. 2021.https://data.police.uk/about/ (accessed 2 Jul 2021).

23 Consumer Data Research Centre. CDRC Residential Mobility Index 2020. London, UK: : UCL 2020.

24 ONS. 2011 Census: Aggregate data (England and Wales). Office for National Statistics 2015. http://www.nationalarchives.gov.uk/doc/open-government-licence/version/2 (accessed 27 Feb 2015).

25 Department for Communities and Local Government. The English Indices of Deprivation 2015. London, UK: 2015.

26 ONS. Council Tax: property attributes (England and Wales): 31 March 2014. 2021. https://www.gov.uk/government/statistics/council-tax-property-attributes (accessed 2 Jul 2021).

27 Jann B. *KMATCH: Stata module module for multivariate-distance and propensity-score matching, including entropy balancing, inverse probability weighting, (coarsened) exact matching, and regression adjustment*. Boston College Department of Economics 2020. https://ideas.repec.org/c/boc/bocode/s458346.html (accessed 20 Jul 2021).

28 Callaway B, Sant'Anna PHC. Difference-in-Differences with multiple time periods. *J Econom* Published Online First: 17 December 2020. doi:10.1016/j.jeconom.2020.12.001

29 ONS. Annual Population Survey (APS) Secure Access UK Data Service. SN: 6721 DOI: 10.5255/UKDA-SN-6721-20. Colchester, UK: : UK Data Service 2021. DOI: 10.5255/UKDA-SN-6721-20 (accessed 2 Jul 2021).

30 Cunningham S. *Causal Inference*. Yale University Press 2021. https://www.degruyter.com/document/doi/10.12987/9780300255881/html (accessed 30 Jun 2021).

31 ONS. The National Statistics Socio-economic classification (NS-SEC). 2021. https://www.ons.gov.uk/methodology/classificationsandstandards/otherclassifications/thenationalstatisticssocioeconomicclassificationnssecrebasedonsoc2010 (accessed 30 Jun 2021).

32 Dolan P, Kavetsos G, Krekel C, *et al.* Quantifying the intangible impact of the Olympics using subjective well-being data. *J Public Econ* 2019;**177**:104043. doi:10.1016/j.jpubeco.2019.07.002

33 Clark C, Smuk M, Cummins S, *et al.* An Olympic Legacy? Did the Urban Regeneration Associated With the London 2012 Olympic Games Influence Adolescent Mental Health? *Am J Epidemiol* 2018;**187**:474–83. doi:10.1093/aje/kwx205

34 Matthay EC, Gottlieb LM, Rehkopf D, *et al.* What to Do When Everything Happens at Once: Analytic Approaches to Estimate the Health Effects of Co-Occurring Social Policies. *Epidemiol Rev* 2022;**43**:33–47. doi:10.1093/epirev/mxab005

35 Schultze A, Nightingale E, Evans D, *et al.* Mortality among Care Home Residents in England during the first and second waves of the COVID-19 pandemic: an observational study of 4.3 million adults over the age of 65. *Lancet Reg Health - Eur* 2022;**14**:100295. doi:10.1016/j.lanepe.2021.100295

For peer review only - http://bmjopen.bmj.com/site/about/guidelines.xhtml

36 White J, Greene G, Farewell D, *et al.* Improving Mental Health Through the Regeneration of Deprived Neighborhoods: A Natural Experiment. *Am J Epidemiol* 2017;**186**:473–80. doi:10.1093/aje/kwx086

37 Singh A, Daniel L, Baker E, *et al.* Housing Disadvantage and Poor Mental Health: A Systematic Review. *Am J Prev Med* 2019;**57**:262–72. doi:10.1016/j.amepre.2019.03.018

38 GLA. Police and crime plan: a safer city for all Londoners. 2017. https://www.london.gov.uk//what-we-do/mayors-office-policing-and-crime-mopac/police-and-crime-plan-safer-city-all-londoners (accessed 23 Jul 2021).

39 Delgado-Rodríguez M, Llorca J. Bias. *J Epidemiol Community Health* 2004;**58**:635–41. doi:10.1136/jech.2003.008466

40 ONS. 2011 Census: Flow Data. [data collection]. UK Data Service. SN: 7713, DOI: 10.5255/UKDA-SN-7713-1. Office for National Statistics 2015.

Figures

Figure 1 Trend in area-level outcomes for never-treated versus treated areas in Greater London, 2011-2019. Treated areas shown from year of initiation onwards. Abbreviations: Small Area Mental Health Index (SAMHI), Antisocial behaviour (ASB), Population (Pop).

Figure 2 Trend in individual-level outcomes for never-treated versus treated areas. Treated areas shown from year of initiation onwards.

Figure 3 Average treatment effect on the treated (ATT) for area-level impacts of selective licencing (SL) on Small Area Mental Health Index (SAMHI), Antisocial behaviour (ASB) calls, and population (Pop) turnover in Greater London, 2011-2019. ASB was ln-transformed and ATT shown as ATT%.

Figure 4 Average treatment effect on the treated (ATT) for individual-level impacts of selective licencing (SL) on self-reported anxiety among private renters in Greater London by year of SL introduction, 2011-2019. Time-varying covariates in PSM Adjusted were: age group, sex, native birth, and occupational class. Abbreviations: Propensity Score Matching (PSM).

For peer review only

For peer review only - http://bmjopen.bmj.com/site/about/guidelines.xhtml

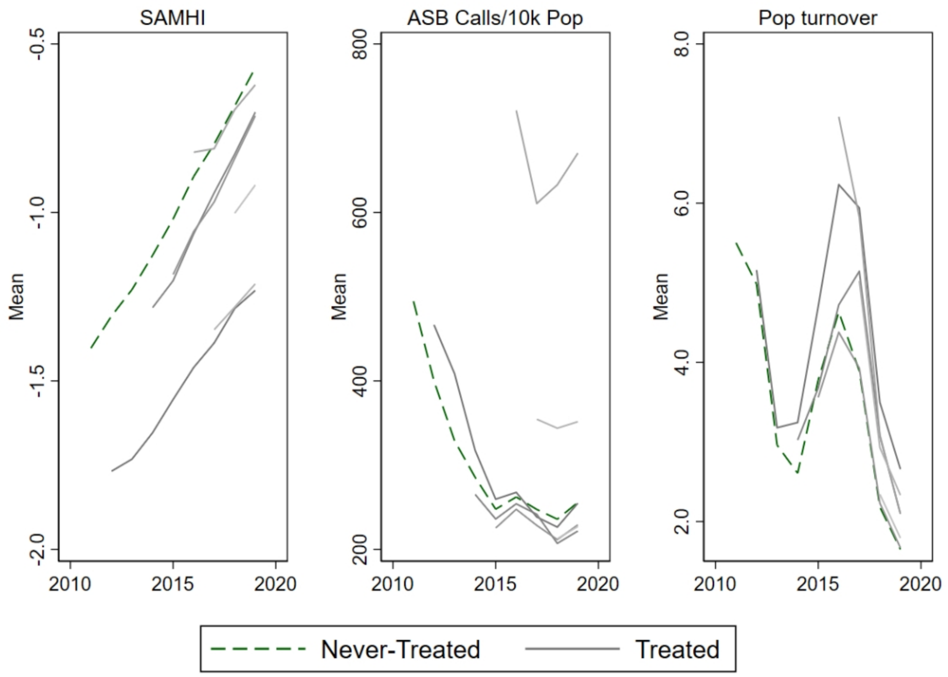

Trend in area-level outcomes for never-treated versus treated areas in Greater London, 2011-2019. Treated areas shown from year of initiation onwards. Abbreviations: Small Area Mental Health Index (SAMHI), Antisocial behaviour (ASB), Population (Pop).

423x307mm (72 x 72 DPI)

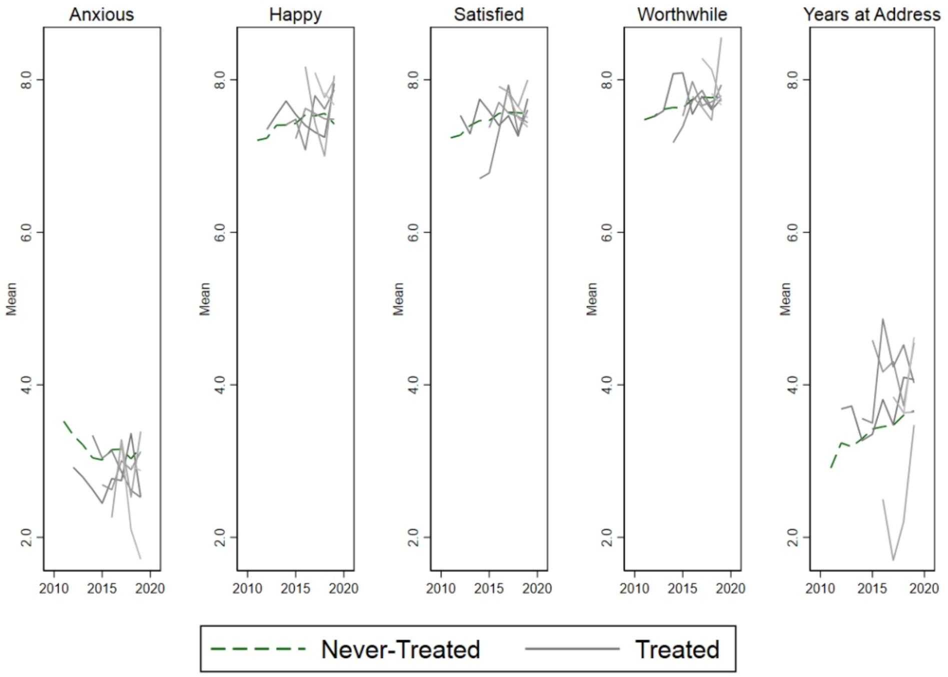

Trend in individual-level outcomes for never-treated versus treated areas. Treated areas shown from year of initiation onwards.

423x307mm (72 x 72 DPI)

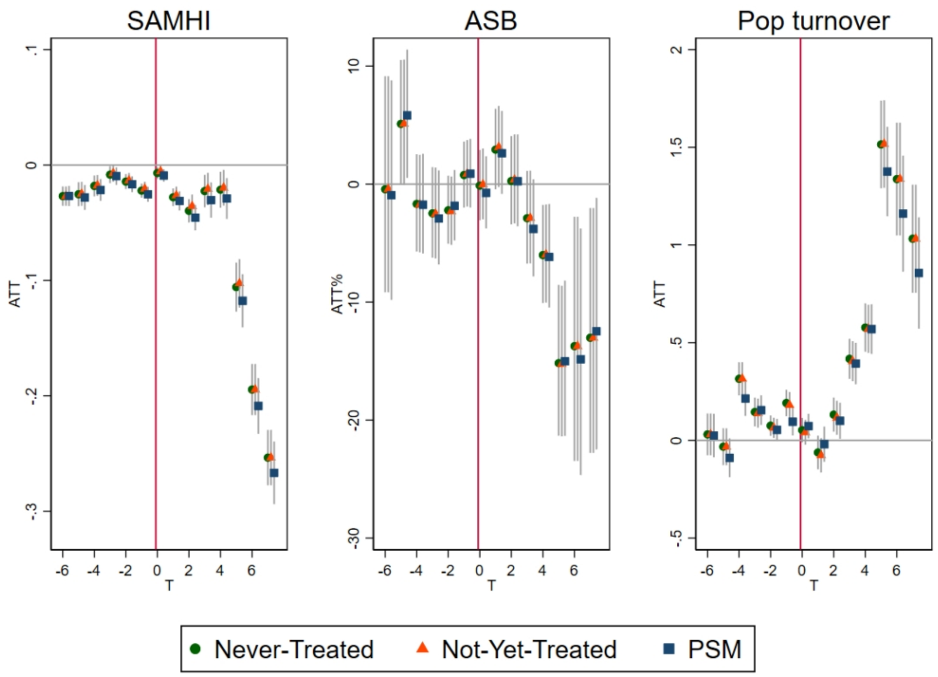

Average treatment effect on the treated (ATT) for area-level impacts of selective licencing (SL) on Small Area Mental Health Index (SAMHI), Antisocial behaviour (ASB) calls, and population (Pop) turnover in Greater London, 2011-2019. ASB was ln-transformed and ATT shown as ATT%.

423x307mm (72 x 72 DPI)

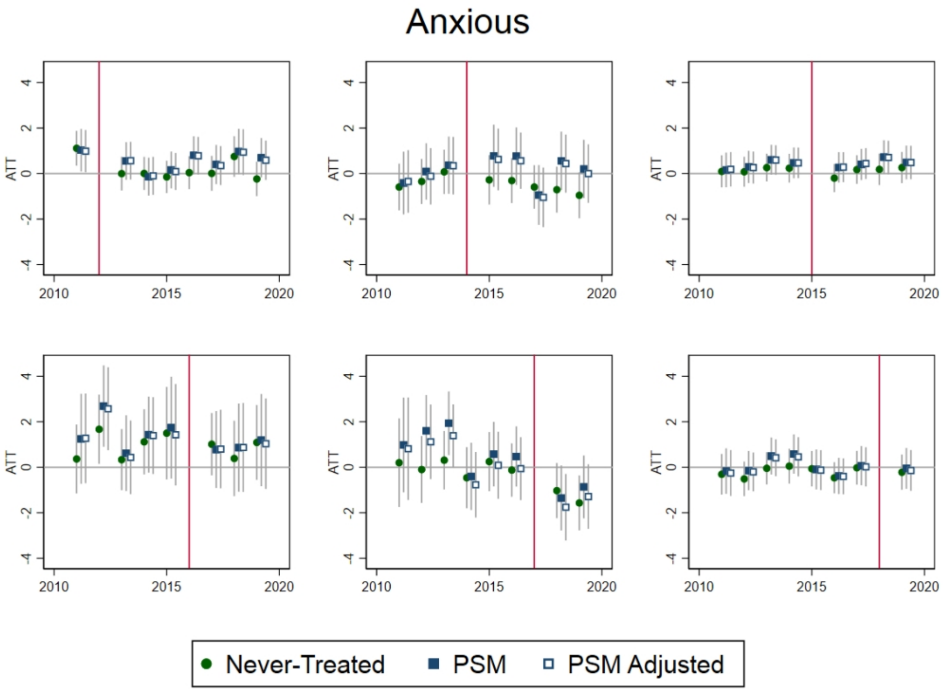

Average treatment effect on the treated (ATT) for individual-level impacts of selective licencing (SL) on self-reported anxiety among private renters in Greater London by year of SL introduction, 2011-2019. Time-varying covariates in PSM Adjusted were: age group, sex, native birth, and occupational class. Abbreviations: Propensity Score Matching (PSM).

423x307mm (72 x 72 DPI)

Supplementary materials

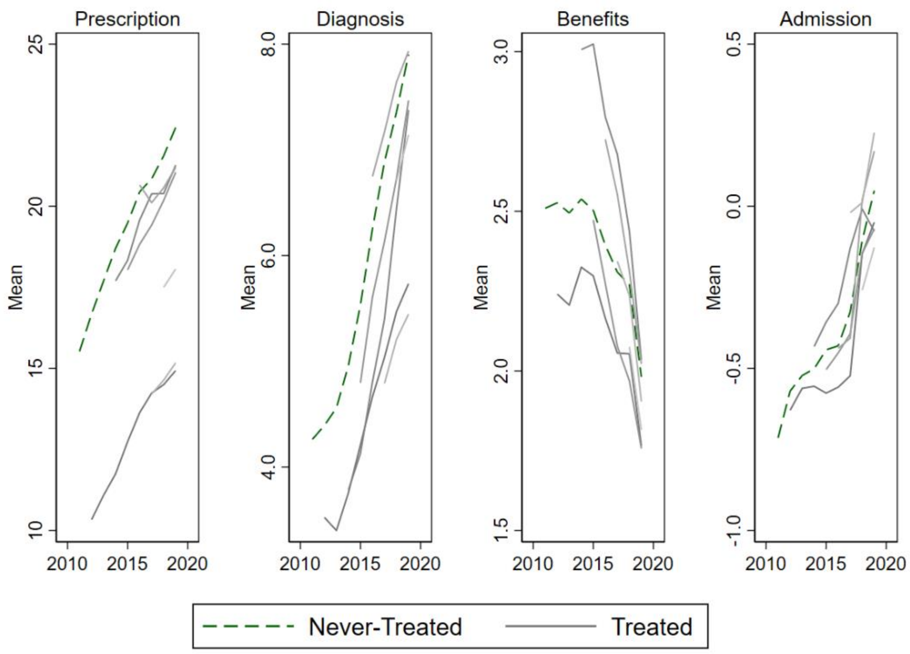

Supplementary Figure 1 Trend in Small Area Mental Health Index (SAMHI) sub-scores (PRESCRIPTION, DIAGNOSIS, BENEFITS, ADMISSION) for never-treated versus treated areas in Greater London, 2011-2019. Treated areas shown from year of initiation onwards..

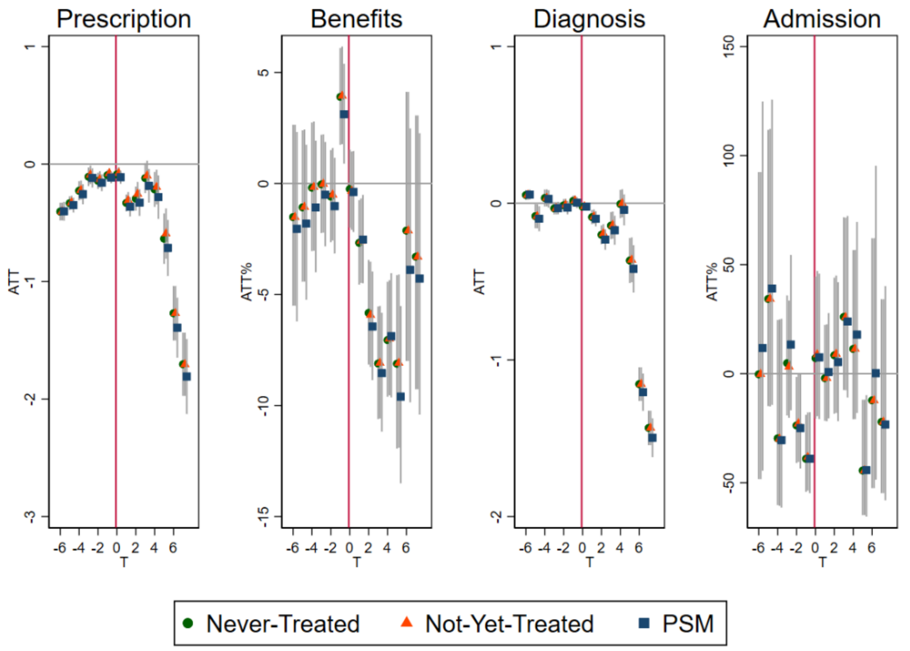

Supplementary Figure 2 Average treatment effect on the treated (ATT) for area-level impacts of selective licencing (SL) on Small Area Mental Health Index (SAMHI) underlying indicators PRESCRIPTION, BENEFITS, DIAGNOSIS, and ADMISSION, in Greater London, 2011-2019. BENEFITS and ADMISSION were ln-transformed and ATT shown as ATT%.

For peer review only - http://bmjopen.bmj.com/site/about/guidelines.xhtml

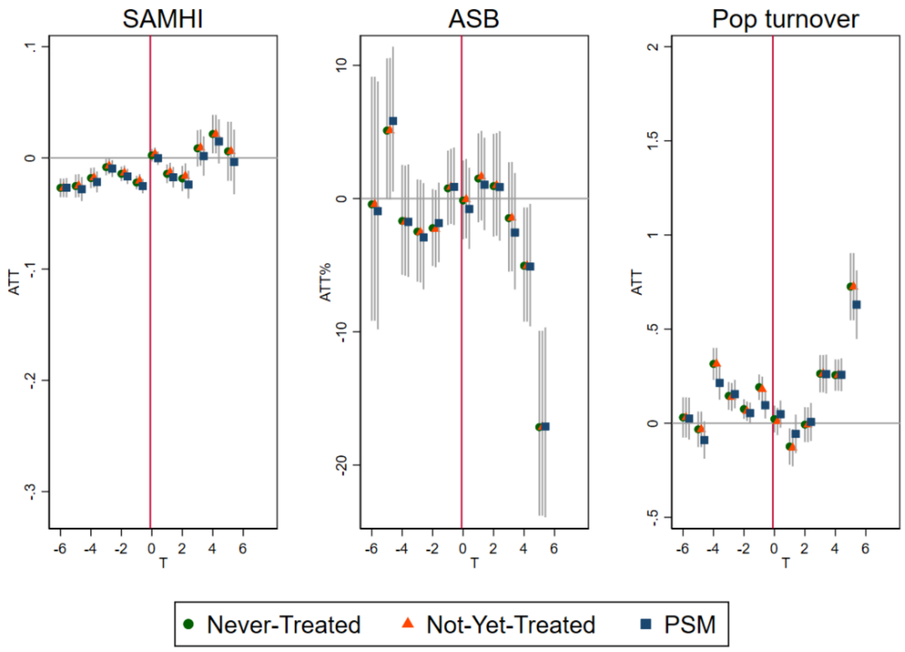

Supplementary Figure 3 Sensitivity check excluding the earliest scheme initiated 2012 (“Olympic”). Average treatment effect on the treated (ATT) for area-level impacts of selective licencing (SL) on Small Area Mental Health Index (SAMHI), Antisocial behaviour (ASB) calls, and population (Pop) turnover in Greater London, 2011-2019. ASB was ln-transformed and ATT shown as ATT%.

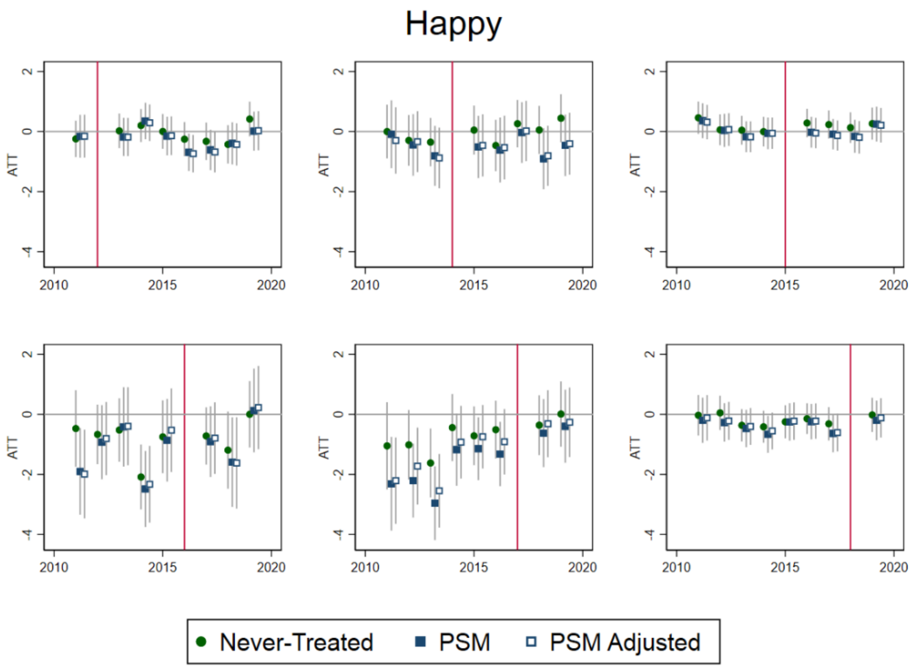

Supplementary Figure 4 Happy. Average treatment effect on the treated (ATT) for individual-level impacts of selective licencing (SL) on self-reported happy score among private renters in Greater London by year of SL introduction, 2011-2019. Time-varying covariates in PSM Adjusted were: age group, sex, native birth, and occupational class. Abbreviations: Propensity Score Matching (PSM).

For peer review only - http://bmjopen.bmj.com/site/about/guidelines.xhtml

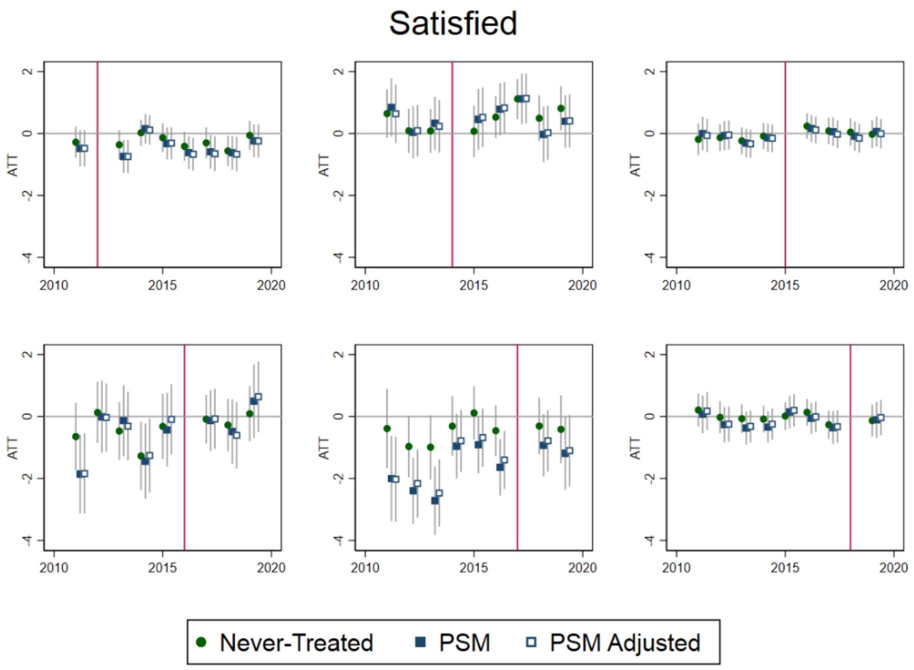

Supplementary Figure 5 Satisfied. Average treatment effect on the treated (ATT) for individual-level impacts of selective licencing (SL) on self-reported satisfied score among private renters in Greater London by year of SL introduction, 2011-2019. Time-varying covariates in PSM Adjusted were: age group, sex, native birth, and occupational class. Abbreviations: Propensity Score Matching (PSM).

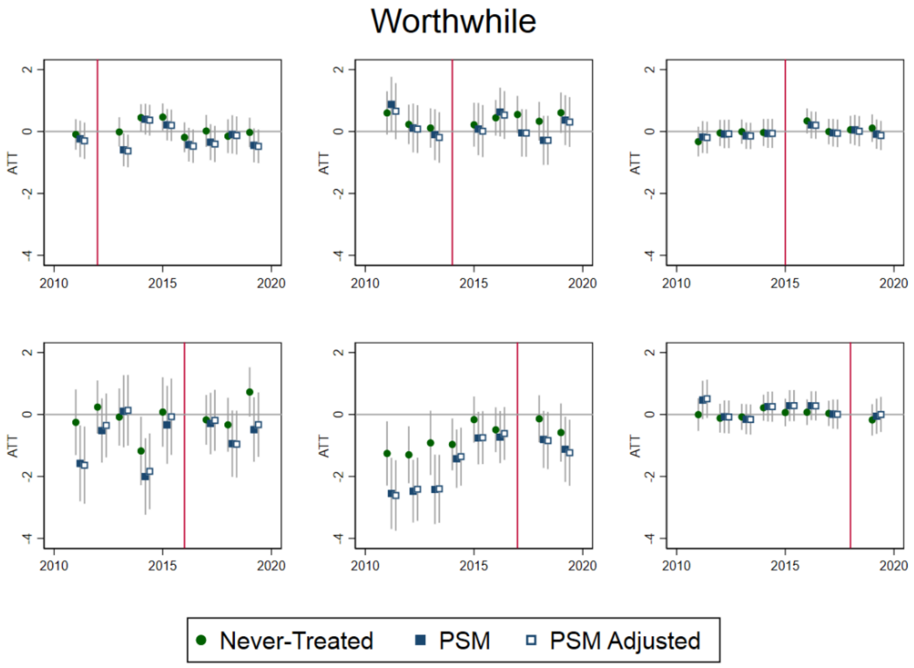

Supplementary Figure 6 Worthwhile. Average treatment effect on the treated (ATT) for individual-level impacts of selective licencing (SL) on self-reported worthwhile score among private renters in Greater London by year of SL introduction, 2011-2019. Time-varying covariates in PSM Adjusted were: age group, sex, native birth, and occupational class. Abbreviations: Propensity Score Matching (PSM).

For peer review only - http://bmjopen.bmj.com/site/about/guidelines.xhtml

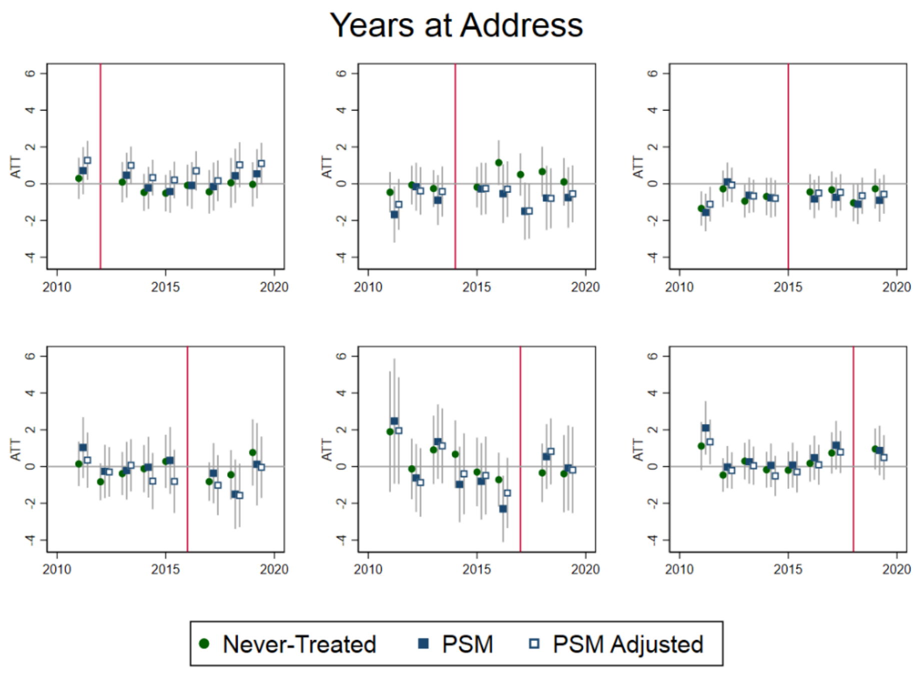

Supplementary Figure 7 Years at address. Average treatment effect on the treated (ATT) for individual-level impacts of selective licencing (SL) on self-reported years at address among private renters in Greater London by year of SL introduction, 2011-2019. Time-varying covariates in PSM Adjusted were: age group, sex, native birth, and occupational class. Abbreviations: Propensity Score Matching (PSM).

Supplementary Table 1 Baseline characteristics for Never-Treated and PSM control areas (LSOA) in Greater London, 2011. PSM controls were used for area-level impacts overall and for each year of treatment initiation, 2012 and 2014-2018, for individual-level impacts. Mean differences tested with a t-test except for Built pre-1945, which was tested with a Chi-square test (alpha=.05). Variables (Data source): Income deprived, Poor housing condition, No central heating, Unaffordable housing (Department for Communities and Local Government 2015b); Built pre-1945 (ONS 2021b); All other (ONS 2015a). Abbreviations: Lower Layer Super output Area (LSOA), Propensity Score Matched (PSM).

**Characteristics**	**All interventions**				
	**Treated**	**Never-Treated**		**PSM**	
	N=921	N=3,582	P-value	N=2,763	P-value
Children <16yr per pop	22.2	19.3	<.001	22.2	**0.839**
Adults 16-59yr per pop	64.1	64.3	**0.436**	63.8	**0.217**
Income deprived per pop	19.3	15.6	<.001	19.2	**0.823**
native birth per pop	60	65.3	<.001	60.6	**0.267**
Private rented%	25.9	23.4	<.001	25.3	**0.288**
Social rented%	22.7	22.5	**0.759**	22.8	**0.881**
Poor housing condition%	22.5	22.4	**0.621**	22.2	**0.298**
No central heating%	2.9	2.8	0.013	2.9	**0.906**
Overcrowded%	23.9	19.7	<.001	23.5	**0.402**
Unaffordable housing measure	2.4	1.7	<.001	2.4	**0.193**
Built pre-1945	-	-	<.001	-	**0.434**
**Characteristics**	**Interventions initiated in 2012**				
	**Treated**	**Never-Treated**		**PSM**	
	N=155	N=3,582	P-value	N=465	P-value
Children <16yr per pop	22.4	19.3	<.001	23.2	**0.153**
Adults 16-59yr per pop	68.2	64.3	<.001	67.7	**0.388**
Income deprived per pop	21.6	15.6	<.001	22.9	**0.15**
native birth per pop	46.5	65.3	<.001	47.4	**0.286**
Private rented%	33.9	23.4	<.001	33.4	**0.66**
Social rented%	28.9	22.5	<.001	31.9	**0.137**

For peer review only - http://bmjopen.bmj.com/site/about/guidelines.xhtml

Poor housing condition%	22.8	22.4	**0.368**	22.7	**0.763**
No central heating%	2.8	2.8	**0.807**	2.8	**0.906**
Overcrowded%	34.9	19.7	<.001	34.8	**0.946**
Unaffordable housing measure	2.8	1.7	<.001	2.9	**0.259**
Built pre-1945	-	-	**0.122**	-	**0.186**
**Characteristics**	**Interventions initiated in 2014**				
	**Treated**	**Never-Treated**		**PSM**	
	N=110	N=3,582	P-value	N=330	P-value
Children <16yr per pop	25.9	19.3	<.001	26.1	**0.753**
Adults 16-59yr per pop	59.9	64.3	<.001	59.9	**0.871**
Income deprived per pop	24.2	15.6	<.001	25.2	**0.354**
native birth per pop	69.5	65.3	0.003	69.2	**0.83**
Private rented%	17.5	23.4	<.001	16.7	**0.361**
Social rented%	33	22.5	<.001	34.8	**0.44**
Poor housing condition%	23.1	22.4	**0.221**	22.3	**0.115**
No central heating%	3	2.8	**0.126**	2.9	**0.49**
Overcrowded%	19.9	19.7	**0.9**	20	**0.86**
Unaffordable housing measure	2.7	1.7	<.001	2.74	**0.793**
Built pre-1945	-	-	**0.498**	-	**0.715**
**Characteristics**	**Interventions initiated in 2015**				
	**Treated**	**Never-Treated**		**PSM**	
	N=394	N=3,582	P-value	N=1,182	P-value
Children <16yr per pop	21.5	19.3	<.001	21.3	**0.468**
Adults 16-59yr per pop	62.8	64.3	<.001	62.2	**0.083**
Income deprived per pop	17.5	15.6	<.001	16.6	**0.073**
native birth per pop	65.6	65.3	**0.664**	67	**0.123**
Private rented%	22.6	23.4	**0.238**	21.3	**0.084**
Social rented%	19.7	22.5	0.008	18.4	**0.196**
Poor housing condition%	22	22.4	**0.25**	21.4	**0.079**
No central heating%	3	2.8	0.005	2.9	**0.255**
Overcrowded%	19.5	19.7	**0.7**	18.4	**0.11**
Unaffordable housing measure	2.2	1.7	<.001	2	**0.063**
Built pre-1945	-	-	0.001	-	**0.723**
**Characteristics**	**Interventions initiated in 2016**				
	**Treated**	**Never-Treated**		**PSM**	
	N=28	N=3,582	P-value	N=84	P-value
Children <16yr per pop	17.8	19.3	**0.106**	16.5	**0.319**
Adults 16-59yr per pop	72.4	64.3	<.001	74	**0.42**
Income deprived per pop	23.5	15.6	<.001	22	**0.443**
native birth per pop	53.6	65.3	<.001	52	**0.413**
Private rented%	33.5	23.4	<.001	36.6	**0.341**
Social rented%	33.3	22.5	0.005	28.8	**0.239**
Poor housing condition%	25.8	22.4	0.003	28	**0.227**
No central heating%	3.1	2.8	**0.226**	3.8	**0.184**
Overcrowded%	35.3	19.7	<.001	36.3	**0.705**
Unaffordable housing measure	3.2	1.7	<.001	2.8	**0.21**
Built pre-1945	-	-	0.024	-	**0.827**
**Characteristics**	**Interventions initiated in 2017**				
	**Treated**	**Never-Treated**		**PSM**	
	N=59	N=3,582	P-value	N=177	P-value
Children <16yr per pop	21.9	19.3	<.001	21.6	**0.79**
Adults 16-59yr per pop	65.5	64.3	**0.219**	66	**0.618**
Income deprived per pop	20.3	15.6	<.001	21.3	**0.371**
native birth per pop	45.4	65.3	<.001	45.5	**0.961**
Private rented%	33.2	23.4	<.001	35.1	**0.345**
Social rented%	18.6	22.5	**0.141**	20.1	**0.547**

For peer review only

For peer review only - http://bmjopen.bmj.com/site/about/guidelines.xhtml

Poor housing condition%	24	22.4	0.048	25.6	**0.148**
No central heating%	2.7	2.8	**0.564**	2.8	**0.701**
Overcrowded%	30.9	19.7	<.001	32.1	**0.407**
Unaffordable housing measure	3.5	1.7	<.001	3.3	**0.245**
Built pre-1945	-	-	0.027	-	**0.404**
**Characteristics**	**Interventions initiated in 2018**				
	**Treated**	**Never-Treated**		**PSM**	
	N=175	N=3,582	P-value	N=525	P-value
Children <16yr per pop	21.9	19.3	<.001	22.1	**0.806**
Adults 16-59yr per pop	64.3	64.3	**0.926**	64	**0.703**
Income deprived per pop	17.2	15.6	0.027	17	**0.787**
native birth per pop	58.8	65.3	<.001	58.8	**0.998**
Private rented%	27.8	23.4	<.001	27.5	**0.833**
Social rented%	17.2	22.5	<.001	17	**0.884**
Poor housing condition%	21.8	22.4	**0.266**	21.7	**0.708**
No central heating%	2.8	2.8	**0.866**	2.8	**0.747**
Overcrowded%	22.3	19.7	0.004	22	**0.785**
Unaffordable housing measure	2.2	1.7	<.001	2.2	**0.865**
Built pre-1945	-	-	0.001	-	**0.955**

For peer review only

For peer review only - http://bmjopen.bmj.com/site/about/guidelines.xhtml

STROBE Statement—Checklist of items that should be included in reports of ***cohort studies***

	Item No	Recommendation	Page No
**Title and abstract**	1	(*a*) Indicate the study's design with a commonly used term in the title or the abstract<br>(*b*) Provide in the abstract an informative and balanced summary of what was done and what was found	1-2
**Introduction**			
Background/rationale	2	Explain the scientific background and rationale for the investigation being reported	3
Objectives	3	State specific objectives, including any prespecified hypotheses	4
**Methods**			
Study design	4	Present key elements of study design early in the paper	4-5
Setting	5	Describe the setting, locations, and relevant dates, including periods of recruitment, exposure, follow-up, and data collection	4-5
Participants	6	(*a*) Give the eligibility criteria, and the sources and methods of selection of participants. Describe methods of follow-up<br>(*b*) For matched studies, give matching criteria and number of exposed and unexposed	4-5
Variables	7	Clearly define all outcomes, exposures, predictors, potential confounders, and effect modifiers. Give diagnostic criteria, if applicable	4-5
Data sources/ measurement	8*	For each variable of interest, give sources of data and details of methods of assessment (measurement). Describe comparability of assessment methods if there is more than one group	4-5
Bias	9	Describe any efforts to address potential sources of bias	6-8
Study size	10	Explain how the study size was arrived at	4-5
Quantitative variables	11	Explain how quantitative variables were handled in the analyses. If applicable, describe which groupings were chosen and why	4-5
Statistical methods	12	(*a*) Describe all statistical methods, including those used to control for confounding<br>(*b*) Describe any methods used to examine subgroups and interactions<br>(*c*) Explain how missing data were addressed<br>(*d*) If applicable, explain how loss to follow-up was addressed<br>(*e*) Describe any sensitivity analyses	4-5
**Results**			
Participants	13*	(a) Report numbers of individuals at each stage of study—eg numbers potentially eligible, examined for eligibility, confirmed eligible, included in the study, completing follow-up, and analysed<br>(b) Give reasons for non-participation at each stage<br>(c) Consider use of a flow diagram	5-6
Descriptive data	14*	(a) Give characteristics of study participants (eg demographic, clinical, social) and information on exposures and potential confounders<br>(b) Indicate number of participants with missing data for each variable of interest<br>(c) Summarise follow-up time (eg, average and total amount)	5-6
Outcome data	15*	Report numbers of outcome events or summary measures over time	5-6

For peer review only - http://bmjopen.bmj.com/site/about/guidelines.xhtml

Main results	16	(*a*) Give unadjusted estimates and, if applicable, confounder-adjusted estimates and their precision (eg, 95% confidence interval). Make clear which confounders were adjusted for and why they were included<br>(*b*) Report category boundaries when continuous variables were categorized<br>(*c*) If relevant, consider translating estimates of relative risk into absolute risk for a meaningful time period	5-6
Other analyses	17	Report other analyses done—eg analyses of subgroups and interactions, and sensitivity analyses	5-6
**Discussion**			
Key results	18	Summarise key results with reference to study objectives	6
Limitations	19	Discuss limitations of the study, taking into account sources of potential bias or imprecision. Discuss both direction and magnitude of any potential bias	6-8
Interpretation	20	Give a cautious overall interpretation of results considering objectives, limitations, multiplicity of analyses, results from similar studies, and other relevant evidence	6-8
Generalisability	21	Discuss the generalisability (external validity) of the study results	6-8
**Other information**			
Funding	22	Give the source of funding and the role of the funders for the present study and, if applicable, for the original study on which the present article is based	1

*Give information separately for exposed and unexposed groups.

Note: An Explanation and Elaboration article discusses each checklist item and gives methodological background and published examples of transparent reporting. The STROBE checklist is best used in conjunction with this article (freely available on the Web sites of PLoS Medicine at http://www.plosmedicine.org/, Annals of Internal Medicine at http://www.annals.org/, and Epidemiology at http://www.epidem.com/). Information on the STROBE Initiative is available at http://www.strobe-statement.org.

For peer review only

For peer review only - http://bmjopen.bmj.com/site/about/guidelines.xhtml

BMJ Open

Impact of selective licencing schemes for private rental housing on mental health and social outcomes in Greater London, England: a natural experiment study

Journal:	*BMJ Open*
Manuscript ID	bmjopen-2022-065747.R1
Article Type:	Original research
Date Submitted by the Author:	01-Nov-2022
Complete List of Authors:	PETERSEN, JAKOB; London School of Hygiene and Tropical Medicine, ; UCL, Geography<br>Alexiou, Alexandros; University of Liverpool Faculty of Health and Life Sciences, Department of Public Health and Policy<br>Brewerton, David; Patient and public involvement representative<br>Cornelsen, Laura; London School of Hygiene and Tropical Medicine,<br>Courtin, Emilie; LSHTM,<br>Cummins, Steven; London School of Hygiene and Tropical Medicine, Public Health, Environments & Society<br>Marks, Dalya; London School of Hygiene & Tropical Medicine, Department of Social and Environmental Health Research<br>Seguin, Maureen ; London School of Hygiene and Tropical Medicine,<br>Stewart, Jill; Middlesex University, School of Science & Technology, Department of Natural Sciences<br>Thompson, Kevin; London Borough of Hackney, Head of Private Sector Housing<br>Egan, Matt; LSHTM, SEHR
**Primary Subject Heading**:	Public health
Secondary Subject Heading:	Epidemiology, Mental health
Keywords:	EPIDEMIOLOGY, MENTAL HEALTH, PUBLIC HEALTH

SCHOLARONE™
Manuscripts

I, the Submitting Author has the right to grant and does grant on behalf of all authors of the Work (as defined in the below author licence), an exclusive licence and/or a non-exclusive licence for contributions from authors who are: i) UK Crown employees; ii) where BMJ has agreed a CC-BY licence shall apply, and/or iii) in accordance with the terms applicable for US Federal Government officers or employees acting as part of their official duties; on a worldwide, perpetual, irrevocable, royalty-free basis to BMJ Publishing Group Ltd ("BMJ") its licensees and where the relevant Journal is co-owned by BMJ to the co-owners of the Journal, to publish the Work in this journal and any other BMJ products and to exploit all rights, as set out in our licence.

The Submitting Author accepts and understands that any supply made under these terms is made by BMJ to the Submitting Author unless you are acting as an employee on behalf of your employer or a postgraduate student of an affiliated institution which is paying any applicable article publishing charge ("APC") for Open Access articles. Where the Submitting Author wishes to make the Work available on an Open Access basis (and intends to pay the relevant APC), the terms of reuse of such Open Access shall be governed by a Creative Commons licence – details of these licences and which Creative Commons licence will apply to this Work are set out in our licence referred to above.

Other than as permitted in any relevant BMJ Author's Self Archiving Policies, I confirm this Work has not been accepted for publication elsewhere, is not being considered for publication elsewhere and does not duplicate material already published. I confirm all authors consent to publication of this Work and authorise the granting of this licence.

For peer review only

Title

Impact of selective licencing schemes for private rental housing on mental health and social outcomes in Greater London, England: a natural experiment study

Authors

Authors [Alphabetical order except first/last]
Jakob Petersen[1]*, PhD, research fellow
Alexiou, Alexandros[2], PhD, research associate
David Brewerton, Patient and Public Involvement representative
Laura Cornelsen[1], PhD, associate professor
Emilie Courtin[1], PhD, assistant professor
Steven Cummins[1], PhD, professor
Dalya Marks[1], PhD, associate professor
Maureen Seguin[1], PhD, research fellow
Jill Stewart[3], PhD, senior lecturer
Kevin Thompson[4], BSc, head of private housing
Matt Egan[1], PhD, professor

Affiliations
1. London School of Hygiene & Tropical Medicine, Public Health, Environments and Society, 15-17 Tavistock Place, London, WC1H 9SH, UK.
2. University of Liverpool, Department of Public Health, Policy and Systems, Waterhouse Building, Block B, Brownlow Street, Liverpool, L69 3GF, UK.
3. Middlesex University, Natural Sciences, The Burroughs, Hendon, London NW4 4BT, UK.
4. London Borough of Hackney, 1 Hillman Street, London, E8 1DY, UK.

*) Corresponding author (JP): jakob.petersen1@lshtm.ac.uk/ jakob.petersen@qmul.ac.uk;

https://orcid.org/0000-0002-6659-7028

For peer review only

Abstract (269/300 words)

Objectives

To assess primary impact of Selective Licencing (SL), an area-based intervention in the private rented housing market, on individual self-reported anxiety and neighbourhood mental health (MHI - mental healthcare index) and secondary impacts on antisocial behaviour (ASB), population turnover, and self-reported wellbeing.

Design

Difference-in-differences (DiD) was used to evaluate effects of SL schemes initiated 2012-2018. 921 intervention areas (Lower Super Output Areas) were matched 3:1 using propensity scores derived from sociodemographic and housing variables (N=3.684 incl. controls). Average Treatment effect on Treated (ATT) was calculated for multiple time period DiD in area-level analyses. Canonical DiD was used for individual-level analysis by year of treatment initiation while adjusting for age, sex, native birth, and occupational class.

Setting

Intervention neighbourhoods and control areas in Greater London, UK, 2011-2019.

Participants

We sampled 4,474 respondents renting privately in intervention areas (N=17,347 incl. controls) in Annual Population Survey and obtained area-level MHI population data.

Interventions

Private landlords in SL areas must obtain a licence from the local authority, allow inspection, and maintain minimum housing standards.

Results

ATT after 5 years was significantly lower for MHI (-7.5%, 95% confidence intervals -5.6;-8.8) than controls. Antidepressant treatment days per population reduced by -5.4% (-3.7;-7.3), mental health benefit receipt by -9.6% (-14;-5.5) and proportion with depression by -12% (-7.7;-16.3). ASB reduced by -15% (-21;-8.2). Population turnover increased by 26.5% (22.1;30.8). Sensitivity analysis suggests overlap with effects of London 2012 Olympic regeneration. No clear patterns were observed for self-reported anxiety.

Conclusions

We found associations between SL and reductions in area-based mental healthcare outcomes and ASB, while population turnover increased. A national evaluation of SL is feasible and necessary.

For peer review only

Article Summary

Strengths and limitations of this study

- This is the first-ever evaluation of mental health and social outcomes of selective licencing schemes.
- The multiple time period DiD design assesses impacts of the staggered area-based intervention over and above a host of other factors that influence mental health and wellbeing.
- A limitation is that it is inherently not possible to eliminate selection bias due to non-random treatment allocation of selective licencing schemes.
- As a limitation, the area-level findings of this study could not be complemented by individual-level data due to data sparsity in the survey sample.

Key words

Social determinants of health; Housing; Public health intervention; Evaluation; Natural experiment; Private Rented Sector

For peer review only - http://bmjopen.bmj.com/site/about/guidelines.xhtml

Introduction

Housing quality affects health [1]. Poor quality homes present numerous environmental risks to residents' health, including risks of injury, physical illnesses linked to cold, damp, and indoor pollution, and risks to mental health and wellbeing [2]. The costs to the English healthcare system attributed to poor housing rivals those associated with hazards such as smoking and alcohol consumption [3,4]; costing an estimated £1.4bn in 2021 [4]. The unequal distribution of poor-quality homes across the population correlates with other social inequalities in health [5].

Housing improvement interventions can have a positive impact on residents' health, including mental health and wellbeing, particularly when targeted at those most in need [2,6–9]. Therefore, strategies for improving population health and health equity often include housing improvement [1,10].

Housing quality improved between 2000 and 2019 in England across all sectors, but conditions are consistently worse in the private rented sector (PRS) compared to owner-occupied and the social rented sector [11]. For instance, the proportion of homes failing to meet the criteria of the Decent Homes Standard in 2019 was 23% in PRS compared to 12% in the social rented sector and 16% for owner occupied homes. The PRS doubled between 2000 and 2019 in tandem with falling affordability of private homes and shrinking of the social housing sector [11].

The need for action to improve PRS quality has been recognised by UK governmental bodies such as the National Audit Office [12] as well as the Chartered Institute for Environmental Health [13]. In 2006, local authorities gained discretionary powers to regulate privately rented homes through 'selective licencing' (SL) schemes under Housing Act 2004 [14]. In SL schemes, landlords in areas targeted by local authorities must pay for a licence, allow inspection, and carry out work necessary to maintain minimum housing standards. Fees are typically around £600 for a 5-year licence. SL schemes can only be implemented following a consultation with local stakeholders and only some local authorities have implemented SL to date [15].

There are very few experimental and long-running studies of the links between housing and health due to lack of acceptability, ethics, treatment blinding, and funding [2,9]. The evidence therefore mainly comes from observational, and often short-term, studies of both individuals and neighbourhoods [7,9]. Although housing improvement interventions have on occasion been implemented as part of a randomised controlled study [9], they are more typically implemented in ways that would require natural experimental impact evaluations.

A systematic review of the effect of housing improvement on health outcomes published in 2013 found the clearest evidence for interventions around thermal comfort, especially if targeted at people with the highest needs (poorer baseline health and/or socio-economic status) [9]. Being able to heat the home economically had positive impacts on health outcomes (general health, mental health, respiratory health, reduced absences from work and school) as well as facilitating better use of indoor space for the residents. In 2019, a systematic review of English-language studies from high-income countries found, in addition to heating, health benefits from improved ventilation, improved water supply, and removal of indoor hazards [2]. Another recent review found evidence that mental health, wellbeing and other outcomes are at risk in the PRS, although the evidence base for interventions that might improve the sector was poor [16].

Initially, Government guidance on SL stated that schemes can be implemented to combat area-level problems such as antisocial behaviour (ASB) [17]. The Housing Act 2004 stipulates that SL can only be implemented as a response to localised problems with low housing demand and persistent ASB [16]. ASB is defined in law as behaviours causing 'harassment, alarm, or distress', which ranges from littering to complaints over rowdy neighbours [18]. New legislation enacted in 2015, however, gave local authorities wider powers to designate areas to SL based on poor housing conditions, high level of migration, deprivation, and crime in addition to the previous conditions [17]. A survey of local authorities in 2019 found poor property conditions closely followed by ASB as the most common reasons for introducing SL. Low demand (vacant housing), deprivation and crime were less commonly cited as reasons for introducing SLs and migration was rarely cited [14].

For peer review only - http://bmjopen.bmj.com/site/about/guidelines.xhtml

A study commissioned by the Department for Levelling Up, Housing and Communities has described how local authorities vary their approach to regulating the PRS. [19] As the legislation allows some flexibility in how SL is implemented, there is scope for local authorities to tailor their SL to the local context and to addressing the reasons for introducing their scheme. An independent review found evidence that local schemes could vary their approach, along with a range of stakeholder views on potential mechanisms by which SL may affect ASB [14]. Although housing improvement interventions can lead to neighbourhood-level improvements [7,9], the mechanisms by which SL may achieve such impacts (incl. on ASB) are complex. SL schemes may include licence conditions that landlords take reasonable action to prevent and reduce ASB. Tenants may face eviction due to ASB and subsequently modify their behaviours, or be evicted. SL may also facilitate joint working across different agencies to tackle underlying issues associated with ASB, or assist policing, or provide training and support to encourage better standards in the sector [14]. We also hypothesise that improved property and positive feelings towards an area may link to reduced ASB. However, unintended impacts of SL, including potential harms, can also be hypothesised. For example, it is possible that costs for licence fees and required improvements are passed on to tenants, and leads to evictions. As a result, households experiencing hardships may be displaced to other localities or face homelessness. We will explore such mechanisms further in a subsequent paper based on qualitative data.

There have not been any systematic attempts to measure the potential impact of SL on mental health, wellbeing, and ASB. This natural experiment study addresses this gap and functions as a feasibility study for a national evaluation of the impacts of SL. This paper primarily evaluates impacts on individual self-reported anxiety and neighbourhood mental healthcare in areas that have implemented SL compared to controls in Greater London. Secondarily, it evaluates self-reported wellbeing outcomes at the individual level, and ASB and population turnover at the area level.

Materials and methods

A protocol paper describing the methodology in more detail has been published previously [20]. This paper concerns the quantitative outcomes of the protocol. The qualitative outcomes are currently being written up in a separate paper by the authors. Separate quantitative and qualitative papers allows for a more detailed descriptions of methods and findings from the two wings of the study.

Patient and public involvement

We consulted two Patient and Public Involvement representatives throughout the project.

Interventions

We obtained details of the spatial and temporal extent of all current and historic SL schemes through Freedom of Information requests (FOI) to all 33 local authorities in Greater London from when first enacted in 2006 to the end of 2019. We included all schemes initiated in or before 2018 in the analyses (Table 1). To standardise the area-based data for analysis, conversion weights were calculated based on the number of 2011 Census enumeration postcodes [21] falling into small intercepts between the de facto geographical unit and the unit of analysis, Lower Layer Super Output Areas 2011 (LSOA; approx. 1,700 average population) [22]. LSOA units that were only partially under treatment (conversion weights >0 and <1) were removed from both the treatment and control pool prior to analysis (N=17 LSOA excluded). Data from two boroughs that introduced street-level schemes (N=279 LSOA excluded), i.e. Hammersmith & Fulham and Southwark, and a single electoral ward that was used as a pilot in Newham (N=9 LSOA excluded) were also excluded.

Table 1 Selective Licencing (SL) schemes in Greater London up until 2018 [Year/Local authority]. Geographies were standardised to fully treated LSOA units. Population estimates are based on Census 2011. APS Private renter interviews in 2011-2019 tabulated by year of treatment initiation. Abbreviations: Annual Population Survey (APS), Lower Layer Super Output Area (LSOA).

For peer review only - http://bmjopen.bmj.com/site/about/guidelines.xhtml

Scheme	LSOA spatial units N	Population 2011 N	Treated private renters Annual Mean (Min;Max)	Total N	Control private renters Annual Mean (Min;Max)	Total N	Treated + Controls Total N
2012 Newham	155	291,351	110 (61;143)	994	298 (175;393)	2,686	3,680
2014 Barking-Dagenham	110	185,911	66 (54;73)	590	104 (83;132)	937	1,527
2015 Brent	23	47,476					
2015 Waltham Forest	144	258,249					
2015 Croydon	220	363,378					
2015 Harrow	7	11,653	156 (116;202)	1,406	549 (428;628)	4,938	6,344
2016 Harrow	6	11,394					
2016 Tower Hamlets	22	38,354	22 (16;35)	200	57 (25;82)	511	711
2017 Ealing	43	77,024					
2017 Redbridge	16	28,789	31 (11;50)	278	135 (74;181)	1,214	1,492
2018 Harrow	14	24,491					
2018 Brent	42	75,793					
2018 Bexley	13	23,499					
2018 Hackney	15	26,366					
2018 Redbridge	91	164,845	112 (74;141)	1,006	287 (225;337)	2,587	3,593
Total	921	1,628,573	-	4,474	-	12,873	17,347

Outcomes – Area-level impacts

Small Area Mental Health Index (SAMHI) scores were obtained by year and small area (LSOA) [23]. SAMHI combines data on mental healthcare from multiple sources into a single index, i.e. National Health Service (NHS) data on z-score standardised mental health-related admission (referred to as ADMISSION, hereinafter), antidepressant treatment days per population (PRESCRIPTION), primary care data on the percentage of the population diagnosed with depression (DIAGNOSIS), and Department for Work and Pensions data on the percentage of population in receipt of mental health-related benefits (BENEFITS). The SAMHI score is proportional to the overall burden on the healthcare system, i.e. an increase signifies a worsening outcome. Each of the underlying SAMHI indicators (ADMISSION, PRESCRIPTION, DIAGNOSIS, BENEFITS) were, according to protocol, studied individually if a positive result was obtained with SAMHI itself.

High levels of ASB is one of the most common reasons for local authorities to implement SL [14], so we assessed the incidence of police-recorded ASB by year and LSOA as a secondary outcome [24]. Data from a population turnover index were studied as a secondary outcome to test an association between SL exposure and moves [25].

The population turnover index data are estimates based on a combination of electoral roll and consumer data (CDRC Residential Mobility Index 2020) [25]. We include the index as a proxy for changes in residential moves. The index is released as a cumulative and the annual proportion of households that will move in the coming year was derived for these analyses. The background for the index is the absence of officially released data other than the decennial censuses. The starting point for the index is the edited electoral roll (i.e. the publicly available version without data on individuals who have opted out for privacy reasons and to avoid direct marketing) complemented with data on names and addresses of consumers collected by commercial data services companies [25].

Statistical methods – Area-level impacts

A Difference-in-Differences (DiD) approach was deployed for the area-level impacts with three different strategies for controls: 1. All never-treated areas, 2. Propensity Score Matched control (PSM) areas (the primary control strategy), and 3. Not-yet-treated areas. The PSM controls were intended as a counterfactual based on measured baseline area characteristics, while the Not-yet-treated controls, a counterfactual for unmeasured characteristics. Local authorities can justify the introduction of SL based on locally held data, e.g. poor housing conditions. This is what we mean by the term unmeasured characteristics in these analyses. Never-treated controls were studied as a check of bias potentially introduced by the matching and trimming of the sample in PSM. The PSM used as far as possible pre-intervention sociodemographic, housing, and neighbourhood characteristics from the

For peer review only

For peer review only - http://bmjopen.bmj.com/site/about/guidelines.xhtml

2011 Census, Indices of Multiple Deprivation, and official dwelling age data (Supplementary Table 1) [26–28]. The matching was carried out with the Stata module KMATCH [29]. The parallel trend assumption was checked visually in the DiD plots.

Homeowners and social renters were by design studied in parallel with private renters for falsifiability checks. SL should only directly affect private renters and any effects detected for private renters could therefore also be challenged by studying not directly affected groups in the same intervention areas. Given the staggered nature of the intervention, a DiD method for comparing multiple time periods were used [30]. The number of intervention LSOA units was 921 and the total number of LSOA in the DiD-PSM analysis was 3,684 (incl. 3 controls per 1 intervention area) (Table 1). The average treatment effect on the treated (ATT) estimated by the DiD was given as ATT% for Ln-transformed indicators (BENEFITS, ADMISSION, ASB), ATT% = -100*(1-exp(ATT)). ATT% was for comparison also calculated for untransformed variables relative to the baseline value.

Outcomes – Individual-level impacts

Data on adult respondents in Annual Population Survey (APS) in England, 2011-2019, were obtained from Office for National Statistics (ONS) [31]. Among these, we identified 4,474 private renters exposed to the intervention (total number of renters incl. controls, N=17,347) (Table 1). The four subjective health and wellbeing questions in APS (aka. ONS4) with scores from 0 to 10 were assessed. The anxiety question was the primary outcome and the other questions on subjective wellbeing (happiness, life satisfaction, whether the things you do in life are worthwhile), secondary outcomes. Data on how long the respondent had lived at the address (asked in categories and recoded to mid-category values for these analyses) were studied at the same time as a proxy of residential stability.

Statistical methods – Individual-level impacts

A canonical DiD approach was deployed for the individual-level impacts by year of treatment initiation in 2012, 2014, and 2015, respectively [32]. Schemes introduced the same year were pooled for statistical efficiency. Three different controls were used: 1. Never-treated, 2. PSM controls, and 3. PSM adjusted for age, sex, native birth, and occupational class [33].

Results

The size of the different SL schemes in terms of fully treated LSOA units, population, and number of private renters captured in the APS data can found in Table 1.

The overall trend in the composite mental healthcare indicator, SAMHI, was a gradual increase in burden during 2011-2019, while antisocial behaviour calls declined sharply in 2011-2015 and then more slowly for most control and treatment groups (Figure 1). Population turnover fluctuated during the study period. The trends for the underlying SAMHI indicators are shown in Supplementary Figure 1.

The trends for the APS outcomes showed a slight improvement with a decline in how anxious the respondent felt the day before the interview and a slight increase for the other subjective wellbeing indicators (happy, satisfied, worthwhile) and years at address. The trends for the different SL schemes by year of treatment initiation were similar yet noisier presumably due to small number issues in the APS sample (Figure 2).

The ATT with PSM controls after 5 years of intervention were significantly different from baseline for all area-based outcomes, SAMHI, antisocial behaviour calls, and population turnover (Table 2, Figure 3). Further analysis of the underlying SAMHI indicators showed similar positive results for antidepressant prescribing, depression diagnosis, and mental health-related benefits, while no clear patterns were seen with mental health-related hospital admission (Supplementary Figure 2). The

For peer review only - http://bmjopen.bmj.com/site/about/guidelines.xhtml

average number of antidepressant treatment days per population in treatment areas at baseline was 13.1. This number reduced by -0.71 days (95% confidence intervals, -0.95 to -0.48) after 5 years of intervention (Table 2), i.e. a -5.4% (-3.7;-7.3) reduction from the baseline in relative terms. Mental health-related benefits were received by 2.4% of the population at baseline and reduced by -9.6% (-14 to -5.5), i.e. -0.23 (-0.13;-0.34) percentage point change in absolute terms. The proportion of the population diagnosed with depression was 3.5% at baseline and reduced by -0.42 percentage points (-0.57 to -0.27), i.e. -12% (-7.7;-16.3) reduction of baseline in relative terms. Antisocial behaviour calls per 10,000 population was 537 at baseline and reduced (i.e. improved) by -15% (-21 to -8.2). Population turnover, as in the proportion of household that will move in the coming year, was 5.2% at baseline and increased by 1.38 percentage points, i.e. 26.5% (22.1;30.8) in relative terms.

A sensitivity check of excluding the sole scheme initiated in 2012 was carried out. Apart from being the earliest London scheme, it also concerned the borough that was centre for the 2012 London Olympics (we here term it the 'Olympic' scheme). The results showed no 5-year results with SAMHI, similar reduction in antisocial behaviour calls, and a more modest increase in population turnover (Supplementary Figure 3).

There were no clear patterns from the individual level analyses of APS data (Figure 4, Supplementary Figures 4-7).

For peer review only

For peer review only - http://bmjopen.bmj.com/site/about/guidelines.xhtml

Table 2 Average Treatment effect on Treated (ATT) for area and individual impacts after 3, 5, and 7 years with PSM controls. ATT given as ATT% for Ln-transformed indicators (Benefits, Admission, ASB). For individual impacts, ATT adjusted for time-varying sociodemographic covariates and relate to the interventions initiated in 2012, 2014, and 2015. ATT values significant at 5% alpha level shown in bold face. Abbreviations: Antisocial Behaviour calls (ASB), Not Applicable (N/A), Propensity Score Matching (PSM).

Indicator	Unit	Baseline mean (2011)		ATT			ATT%
		Never-Treated	Treated	3-year	5-year	7-year	5-year
Area impacts – Interventions initiated 2012-2018							
SAMHI	Index score	-1.4	-1.6	**-.03 (-.05;-.02)**	**-.12 (-.14;-.09)**	**-.27 (-.29;-.24)**	**-7.5% (-5.6;-8.8)**
-Prescription	Antidepressant treatment	15.5	13.1	**-.19 (-.33;-.04)**	**-.71 (-.95;-.48)**	**-1.81 (-2.13;-1.49)**	**-5.4% (-3.7;-7.3)**
-Benefits	%pop	2.5	2.4	**-8.5% (-11;-5.8)**	**-9.6% (-14;-5.5)**	-4.3% (-10;2.3)	**-9.6% (-14;-5.5)**
-Diagnosis	%pop	4.3	3.5	**-.17 (-.26;-.08)**	**-.42 (-.57;-.27)**	**-1.5 (-1.62;-1.37)**	**-12% (-7.7;-16.3)**
-Admission	z-score	-.71	-.54	24% (-11;72)	**-44% (-66;-9.9)**	-23% (-58;40)	**-44% (-66;-9.9)**
ASB	Calls per 10k pop	495	537	-3.8% (-7.8;.41)	**-15% (-21;-8.2)**	**-12% (-22;-1.2)**	**-15% (-21;-8.2)**
Pop turnover	%households moving	5.5	5.2	**0.39 (.29;.5)**	**1.38 (1.15;1.6)**	**0.86 (0.57;1.14)**	**26.5% (22.1;30.8)**
Individual impacts – Interventions initiated 2012							
Anxious	0-10 scale	3.5	4.2	0.09 (-.72;.9)	0.35 (-.5;1.21)	0.59 (-.28;1.45)	8.3% (-11.9;5)
Happy	0-10 scale	7.2	7.1	-.14 (-.78;.5)	**-.68 (-1.36;-.005)**	0.03 (-.62;.68)	**-9.6% (-19.2;-0.1)**
Satisfied	0-10 scale	7.2	7.2	-.31 (-.83;.2)	**-.65 (-1.2;-.01)**	-.24 (-.78;.29)	**-9% (-16.7;-0.1)**
Worthwhile	0-10 scale	7.5	7.4	0.2 (-.3;.7)	-.4 (-.99;.18)	-.48 (-1.02;.06)	-5.4% (-13.4;2.4)
Years at address	Years	2.9	3.6	0.2 (-.79;1.12)	0.16 (-.95;1.27)	1.1 (-.03;2.23)	4.4% (-26.4;35.3)
Individual impacts – Intervention initiated 2014							
Anxious	0-10 scale	3.5	3.2	-1.05 (-2.35;.26)	-.001 (-1.29;1.28)	N/A	0% (-40.3;40)
Happy	0-10 scale	7.2	7.2	0.02 (-.99;1.02)	-.41 (-1.44;.63)	N/A	-5.7% (-20;8.8)
Satisfied	0-10 scale	7.2	7.1	**1.13 (.32;1.93)**	0.4 (-.44;1.25)	N/A	5.6% (-6.2;17.6)
Worthwhile	0-10 scale	7.5	7.6	-.05 (-.82;.71)	0.31 (-.5;1.11)	N/A	4.1% (-6.6;14.6)
Years at address	Years	2.9	2.7	-1.48 (-2.99;.03)	-.55 (-2.01;1)	N/A	-20.4% (-74.4;37)
Individual impacts – Intervention initiated 2015							
Anxious	0-10 scale	3.5	3.3	0.71 (-.03;1.44)	N/A	N/A	N/A
Happy	0-10 scale	7.2	7.5	-.19 (-.73;.35)	N/A	N/A	N/A
Satisfied	0-10 scale	7.2	7	-.15 (-.62;.31)	N/A	N/A	N/A
Worthwhile	0-10 scale	7.5	7	0.01 (-.48;.5)	N/A	N/A	N/A
Years at address	Years	2.9	2.7	-.65 (-1.64;.33)	N/A	N/A	N/A

For peer review only

For peer review only - http://bmjopen.bmj.com/site/about/guidelines.xhtml

Discussion

The study found improvements in area-based mental health outcomes and antisocial behaviour calls (ASB), while population turnover increased. Conversely, the results for self-reported anxiety and other individual-level indicators were inconclusive due to the small sample size of the APS data.

The results indicate potential benefits of SL schemes beyond their 5-year cycle, especially for reduction of ABS. We cannot exclude that at least part of the change could be due to gentrification and we saw an increase in population turnover to suggest this. Future quantitative studies of area-based impacts should therefore assess whether gentrification effects can be ruled out. Several mechanisms could potentially be at play. SL may encourage better practice amongst landlords and lead to improvements that may be sustained. Alternatively, SL may result in more landlords selling their properties rather than facing the increased cost burden, unregulated rentals, passing costs onto tenants through rent increases, and evicting tenants with ASB behaviours with the opportunity to increase rents in high-demand areas. These hypothesised explanations are not mutually exclusive. Furthermore, the mechanisms at play may vary by scheme given differences in local context and given that the legislation allows for some flexibility in local delivery.

An interesting feature of these findings is that some of the changes in outcomes occurred before the completion of the 5-year licencing periods. This suggests the possibility that SL schemes may have impacts prior to full implementation. This could be important, as levels of enforcement may vary across London schemes: while there has not been a robust evaluation of this issue, the website www.londonpropertylicencing.co.uk provides some information on varying levels of enforcement based on periodic data requests from London local authorities [15].

These first findings may be confounded by the fact that the earliest scheme overlapped with urban regeneration projects in connection with the 2012 London Olympics. A sensitivity check excluding the 'Olympic' scheme (Newham) did not show any reduction in the main area-based mental healthcare indicator, SAMHI. There was, however, a similar reduction in ASB and a more modest increase in population turnover after five years (both statistically significant). Studies of the impacts of the Olympic event itself and its legacy have notably been mixed. A telephone survey of residents in London, Berlin, and Paris in 2011-2013 found a short-lived increase in subjective wellbeing for Londoners during the event [34]. A longitudinal cohort study of adolescents and their families living close to the Olympic site compared to those living further away found no changes in self-reported health behaviours or health outcomes (including subjective wellbeing) from before to 18 months after the event [35]. Co-occurring policies are a potential threat to the validity of our estimates [36]. Future research should therefore repeat our analysis when longer time series are available and more schemes can be studied in London and nationally to disentangle the effects of SL from the long-term effects of the urban regeneration such as those surrounding the London Olympics.

In this study, we defined mental health broadly with indicators ranging from self-reported wellbeing to mental health hospital admission. It is clear that the social surveys that cover subjective wellbeing are typically not designed for sub-regional analysis. Administrative or routinely collected data are, on the other hand, more scalable, yet only capture the more extreme end of the mental health scale, and often very hard to access for researchers due to information governance strictures. Recent developments triggered by the COVID19 pandemic however have opened up new opportunities for secure data linkage at patient address level [37]. This development is promising for the evaluation of housing policies such as SL.

A 10-year natural experiment study of healthcare service use in social housing residents age 60+ years in the UK found that those who received improvements to their kitchens, bathrooms or front doors, among other kinds of improvement, presented less often with common mental health disorders than those who did not receive these improvements [8]. A 5-year study (GoWell) of the impact of housing

For peer review only - http://bmjopen.bmj.com/site/about/guidelines.xhtml

improvements on self-reported mental health and wellbeing among social housing residents found additional positive effects of renewing fabric works, i.e. carpets, curtains, and blinds [6]. The GoWell study also found a positive correlation between self-reported mental health and wellbeing among social housing residents and urban regeneration spending, which locally could cover internal housing, external housing, neighbourhoods, as well as community project investments. It was the residents with the highest needs, who resided in the worst housing in the most rundown neighbourhoods, receiving the highest urban regeneration investment, who ultimately showed the greatest improvements in self-reported mental health [7]. Another UK natural experiment study of urban regeneration found positive effects for residents' mental health [38]. These studies support the link between housing improvement and mental health and wellbeing suggested by the present study.

A recent systematic review on housing and health reported randomised controlled trial evidence about mental health benefits for both children and adults in relation to improvements of heating and ventilation [2]. Another recent systematic review of earlier housing disadvantage and poor mental health outcomes reported clear correlations, but also called for more studies to elucidate mechanisms [39]. Another review identified PRS as a growing yet overlooked sector with wide-ranging needs including mental health needs [16]. The review also acknowledged a current lack of evidence about effective interventions. Taken together, the reviews highlight a need for more and better evidence of social polices aiming to improve housing quality including in PRS.

Reduction of ASB [18] is considered a key objective for the policing of London based on consultation and social surveys on the perception of crime [40]. It is common for local authorities to use reduction of ASB as a justification for SL [14] Interestingly, we found that ASB reduced after 4-5 years of SL – even when we excluded the 'Olympic' scheme. Further studies should examine the reasons for the ASB calls, e.g. whether the calls concern neighbours.

A strength of the study is our use of the DiD design, which assesses impacts over and above a host of other factors that influence mental health and wellbeing. In addition, the multiple time period comparison DiD summarises the effect of a staggered intervention such as SL in a single analysis [30]. This step also enables Not-yet treated as control of unmeasured factors associated with treatment allocation. Never-treated controls were included should true effects be masked by overmatching in the PSM. Reassuringly, the different controls generally yielded similar results in this study.

The area-level findings should be backed up by individual-level findings specific to private renters and free of ecological bias [41]. In this case, we found that the APS sample data were too sparsely populated to create robust panel units over time and that many of the smaller schemes therefore could not be properly assessed. We instead deployed a canonical DiD approach and analysed SL by year of treatment initiation. The results were however inconclusive due the large variation associated with small sample size. Future studies should include data at the national level to reach higher numbers.

A limitation of the study is that while physical housing conditions is a key factor in the logic model linking SL to more distant outcomes such as mental health and wellbeing, no adequate data were available to the authors at this point. We did consider national surveys such as English Housing Survey but assessed them too small for robust analysis, given the relatively sparse coverage of SL to date. We aim to address the important role of physical housing conditions in future studies, e.g. by exploiting data from Energy Performance of Buildings Register or by linking administrative data on housing tenure to administrative healthcare data. We essentially call for more high-quality, data with sufficient temporal and spatial granularity to enable the timely evaluation of housing policies and their impact on both properties, people, and localities.

We also call for a register of private rented properties and landlords to facilitate improved monitoring, evaluation and regulation of this sector. A recent UK government policy paper, *A fairer private rented*

For peer review only - http://bmjopen.bmj.com/site/about/guidelines.xhtml

sector, has proposed a 'Property Portal', with landlords legally required to register their property on the portal [42].

This study is to our knowledge the first to use SAMHI [23] and CDRC Residential Mobility index [25] in an evaluation of an area-based policy such as SL. There was much higher precision in the SAMHI sub-scores, PRESCRIPTION and DIAGNOSIS, than in BENEFITS and ADMISSION. The results with ADMISSION were particularly unprecise and variable. CDRC Residential Mobility index provides yearly estimates of moves, whereas the 'gold standard', the Census flow data, in contrast are only released every ten years [43]. The trend in annual proportion of households that will move in the coming year showed a great deal of fluctuation in itself. Due to the DiD design of this study, 'global' fluctuations are in themselves not prohibitive for an evaluation of an area-based intervention. Future releases should nonetheless examine whether the fluctuations can be explained.

The PSM used as far as possible pre-intervention sociodemographic and housing variables. It is possible that the matching could produce a more realistic counterfactual if more pre-intervention data relevant to treatment allocation and/or outcome risk factors become available in the future.

Conclusions

We found early indications of a reduction in area-based mental health outcomes and ASB, while population turnover increased. Results from the individual-level analysis of APS data were inconclusive; possibly due to sample size issues. Longer time series are needed to disentangle SL from Olympic regeneration. Further studies specific to private renters and gentrification effects are needed. Overall, we argue that a national evaluation of SL is feasible and necessary.

Contributorship
All authors contributed to the conception, study design, data interpretation, and approved the submitted version (JP, AA, DB, LC, EC, SC, DM, MS, JS, KT, ME). JP contributed to data acquisition, data analysis, and drafted the first manuscript.

Competing interests
The investigators declare no competing interests of financial nature and, as a whole, counts both homeowners and tenants.

Funding statement
This study is funded by the National Institute for Health Research (NIHR) School for Public Health Research (SPHR) (Grant Reference Number PD-SPH-2015). The views expressed are those of the authors and not necessarily those of the NIHR or the Department of Health and Social Care.

Data sharing
The data supporting the findings of this study were obtained under licence and as such not available to other researchers. The data are, however, available from Office for National Statistics subject to ethical and scientific approval. This work was produced using statistical data from ONS [Annual Population Survey]. The use of the ONS statistical data in this work does not imply the endorsement of the ONS in relation to the interpretation or analysis of the statistical data. This work uses research datasets which may not exactly reproduce National Statistics aggregates.

Ethics approval

Ethical approval was obtained from London School of Hygiene and Tropical Medicine's Ethics Committee (reference number 26481) and London Borough of Hackney.

References

For peer review only - http://bmjopen.bmj.com/site/about/guidelines.xhtml

For peer review only

1 WHO. WHO Housing and health guidelines. Geneva, Switzerland: 2018. https://www.who.int/publications-detail-redirect/9789241550376 (accessed 21 Jul 2021).

2 Ige J, Pilkington P, Orme J, *et al.* The relationship between buildings and health: a systematic review. *J Public Health* 2019;**41**:e121–32. doi:10.1093/pubmed/fdy138

3 Roys M, Nicol S, Garrett H, *et al. The full cost of poor housing*. Bracknell, UK: : BRE & IHS Global Ltd 2016.

4 Garrett H, Mackay M, Nicol S, *et al.* The cost of poor housing in England. Watford, UK: : BRE Group 2021.

5 Gibson M, Petticrew M, Bambra C, *et al.* Housing and health inequalities: a synthesis of systematic reviews of interventions aimed at different pathways linking housing and health. *Health Place* 2011;**17**:175–84. doi:10.1016/j.healthplace.2010.09.011

6 Curl A, Kearns A, Mason P, *et al.* Physical and mental health outcomes following housing improvements: evidence from the GoWell study. *J Epidemiol Community Health* 2015;**69**:12–9. doi:10.1136/jech-2014-204064

7 Egan M, Kearns A, Katikireddi SV, *et al.* Proportionate universalism in practice? A quasi-experimental study (GoWell) of a UK neighbourhood renewal programme's impact on health inequalities. *Soc Sci Med 1982* 2016;**152**:41–9. doi:10.1016/j.socscimed.2016.01.026

8 Rodgers SE, Bailey R, Johnson R, *et al.* Health impact, and economic value, of meeting housing quality standards: a retrospective longitudinal data linkage study. *Public Health Res* 2018;**6**:1–104. doi:10.3310/phr06080

9 Thomson H, Thomas S, Sellstrom E, *et al.* Housing improvements for health and associated socio-economic outcomes. *Cochrane Database Syst Rev* 2013;:CD008657. doi:10.1002/14651858.CD008657.pub2

10 Marmot M, Allen J, Boyce T, *et al.* Health Equity in England: The Marmot Review 10 Years On. Health Found. 2020.https://www.health.org.uk/publications/reports/the-marmot-review-10-years-on (accessed 10 Jul 2020).

11 Ministry of Housing, Communities and Local Government. English Housing Survey: headline report 2019-2020. London, UK: 2020. https://assets.publishing.service.gov.uk/government/uploads/system/uploads/attachment_data/file/945013/2019-20_EHS_Headline_Report.pdf (accessed 30 Mar 2021).

12 National Audit Office. Regulation of private renting. London, UK: : National Audit Office 2021.

13 Chartered Institute of Environmental Health. A National Registration Scheme for the private rented sector - Parliamentary Briefing 10 July 2019. London, UK: : Chartered Institute of Environmental Health 2019.

14 Lawrence S, Wilson P. An independent review of the use and effectiveness of selective licensing. London, UK: : Ministry of Housing, Communities and Local Government 2019. https://www.gov.uk/government/publications/selective-licensing-review (accessed 30 Mar 2021).

15 Dawson H, Tacagni R. Housing Act 2004 property licencing schemes. In: *Stewart, J. & Moffatt, R. (Eds.) Regulating the Privately Rented Housing Sector*. London, UK: : Routledge 2022.

For peer review only - http://bmjopen.bmj.com/site/about/guidelines.xhtml

16 Harris J, McKee K. Health and wellbeing in the private rented sector Part 1: Literature review. Glasgow: : UK Collaborative Centre for Housing Evidence 2021. https://housingevidence.ac.uk/publications/health-and-wellbeing-in-the-private-rented-sector-part-1-literature-review/ (accessed 10 Nov 2021).

17 Department for Communities and Local Government. Selective licensing in the private rented sector: a guide for local authorities. London, UK: 2015. https://www.gov.uk/government/publications/selective-licensing-in-the-private-rented-sector-a-guide-for-local-authorities (accessed 30 Mar 2021).

18 HM Government. Anti-social Behaviour, Crime and Policing Act 2014. Statute Law Database 2014. https://www.legislation.gov.uk/ukpga/2014/12/contents (accessed 23 Jul 2021).

19 Department for Levelling Up, Housing, and Communities. Research and analysis of Local authority enforcement in the private rented sector: headline report. London, UK: 2022. https://www.gov.uk/government/publications/local-authority-enforcement-in-the-private-rented-sector-headline-report/local-authority-enforcement-in-the-private-rented-sector-headline-report

20 Petersen J, Seguin M, Alexiou A, *et al.* Assessing the impact of selective licencing schemes for private rental housing on mental health and well-being: protocol for a mixed-method natural experiment study in Greater London, UK. *BMJ Open* 2022;**12**:e057711. doi:10.1136/bmjopen-2021-057711

21 ONS. Postcode Headcounts and Household Estimates - 2011 Census. Nomis - Off. Labour Mark. Stat. 2021.https://www.nomisweb.co.uk/census/2011/postcode_headcounts_and_household_estimates (accessed 21 Jul 2021).

22 Simpson L, Yu A. Public access to conversion of data between geographies, with multiple look up tables derived from a postal directory. *Comput Environ Urban Syst* 2003;**27**:283–307. doi:10.1016/S0198-9715(02)00018-2

23 Daras K, Barr B. Small Area Mental Health Index (SAMHI) 2.0, Place-based Longitudinal Data Resource. 2020. https://pldr.org/dataset/2noyv/small-area-mental-health-index-samhi (accessed 2 Jul 2021).

24 Police.uk. data.police.uk. 2021.https://data.police.uk/about/ (accessed 2 Jul 2021).

25 Consumer Data Research Centre. CDRC Residential Mobility Index 2020. London, UK: : UCL 2020.

26 ONS. 2011 Census: Aggregate data (England and Wales). Office for National Statistics 2015. http://www.nationalarchives.gov.uk/doc/open-government-licence/version/2 (accessed 27 Feb 2015).

27 Department for Communities and Local Government. The English Indices of Deprivation 2015. London, UK: 2015.

28 ONS. Council Tax: property attributes (England and Wales): 31 March 2014. 2021. https://www.gov.uk/government/statistics/council-tax-property-attributes (accessed 2 Jul 2021).

29 Jann B. *KMATCH: Stata module module for multivariate-distance and propensity-score matching, including entropy balancing, inverse probability weighting, (coarsened) exact matching, and regression adjustment*. Boston College Department of Economics 2020. https://ideas.repec.org/c/boc/bocode/s458346.html (accessed 20 Jul 2021).

For peer review only - http://bmjopen.bmj.com/site/about/guidelines.xhtml

30 Callaway B, Sant'Anna PHC. Difference-in-Differences with multiple time periods. *J Econom* Published Online First: 17 December 2020. doi:10.1016/j.jeconom.2020.12.001

31 ONS. Annual Population Survey (APS) Secure Access UK Data Service. SN: 6721 DOI: 10.5255/UKDA-SN-6721-20. Colchester, UK: : UK Data Service 2021. DOI: 10.5255/UKDA-SN-6721-20 (accessed 2 Jul 2021).

32 Cunningham S. *Causal Inference*. Yale University Press 2021. https://www.degruyter.com/document/doi/10.12987/9780300255881/html (accessed 30 Jun 2021).

33 ONS. The National Statistics Socio-economic classification (NS-SEC). 2021. https://www.ons.gov.uk/methodology/classificationsandstandards/otherclassifications/thenationalstatisticssocioeconomicclassificationnssecrebasedonsoc2010 (accessed 30 Jun 2021).

34 Dolan P, Kavetsos G, Krekel C, *et al.* Quantifying the intangible impact of the Olympics using subjective well-being data. *J Public Econ* 2019;**177**:104043. doi:10.1016/j.jpubeco.2019.07.002

35 Clark C, Smuk M, Cummins S, *et al.* An Olympic Legacy? Did the Urban Regeneration Associated With the London 2012 Olympic Games Influence Adolescent Mental Health? *Am J Epidemiol* 2018;**187**:474–83. doi:10.1093/aje/kwx205

36 Matthay EC, Gottlieb LM, Rehkopf D, *et al.* What to Do When Everything Happens at Once: Analytic Approaches to Estimate the Health Effects of Co-Occurring Social Policies. *Epidemiol Rev* 2022;**43**:33–47. doi:10.1093/epirev/mxab005

37 Schultze A, Nightingale E, Evans D, *et al.* Mortality among Care Home Residents in England during the first and second waves of the COVID-19 pandemic: an observational study of 4.3 million adults over the age of 65. *Lancet Reg Health - Eur* 2022;**14**:100295. doi:10.1016/j.lanepe.2021.100295

38 White J, Greene G, Farewell D, *et al.* Improving Mental Health Through the Regeneration of Deprived Neighborhoods: A Natural Experiment. *Am J Epidemiol* 2017;**186**:473–80. doi:10.1093/aje/kwx086

39 Singh A, Daniel L, Baker E, *et al.* Housing Disadvantage and Poor Mental Health: A Systematic Review. *Am J Prev Med* 2019;**57**:262–72. doi:10.1016/j.amepre.2019.03.018

40 GLA. Police and crime plan: a safer city for all Londoners. 2017. https://www.london.gov.uk//what-we-do/mayors-office-policing-and-crime-mopac/police-and-crime-plan-safer-city-all-londoners (accessed 23 Jul 2021).

41 Delgado-Rodríguez M, Llorca J. Bias. *J Epidemiol Community Health* 2004;**58**:635–41. doi:10.1136/jech.2003.008466

42 Department for Levelling Up, Housing, and Communities. Policy paper: A fairer private rented sector. London, UK: 2022. https://www.gov.uk/government/publications/a-fairer-private-rented-sector/a-fairer-private-rented-sector

43 ONS. 2011 Census: Flow Data. [data collection]. UK Data Service. SN: 7713, DOI: 10.5255/UKDA-SN-7713-1. Office for National Statistics 2015.

Figures

For peer review only - http://bmjopen.bmj.com/site/about/guidelines.xhtml

Figure 1 Trend in area-level outcomes for never-treated versus treated areas in Greater London, 2011-2019. Treated areas shown from year of initiation onwards. Abbreviations: Small Area Mental Health Index (SAMHI), Antisocial behaviour (ASB), Population (Pop).

Figure 2 Trend in individual-level outcomes for never-treated versus treated areas. Treated areas shown from year of initiation onwards.

Figure 3 Average treatment effect on the treated (ATT) for area-level impacts of selective licencing (SL) on Small Area Mental Health Index (SAMHI), Antisocial behaviour (ASB) calls, and population (Pop) turnover in Greater London, 2011-2019. ASB was ln-transformed and ATT shown as ATT%.

Figure 4 Average treatment effect on the treated (ATT) for individual-level impacts of selective licencing (SL) on self-reported anxiety among private renters in Greater London by year of SL introduction, 2011-2019. Time-varying covariates in PSM Adjusted were: age group, sex, native birth, and occupational class. Abbreviations: Propensity Score Matching (PSM).

For peer review only

For peer review only - http://bmjopen.bmj.com/site/about/guidelines.xhtml

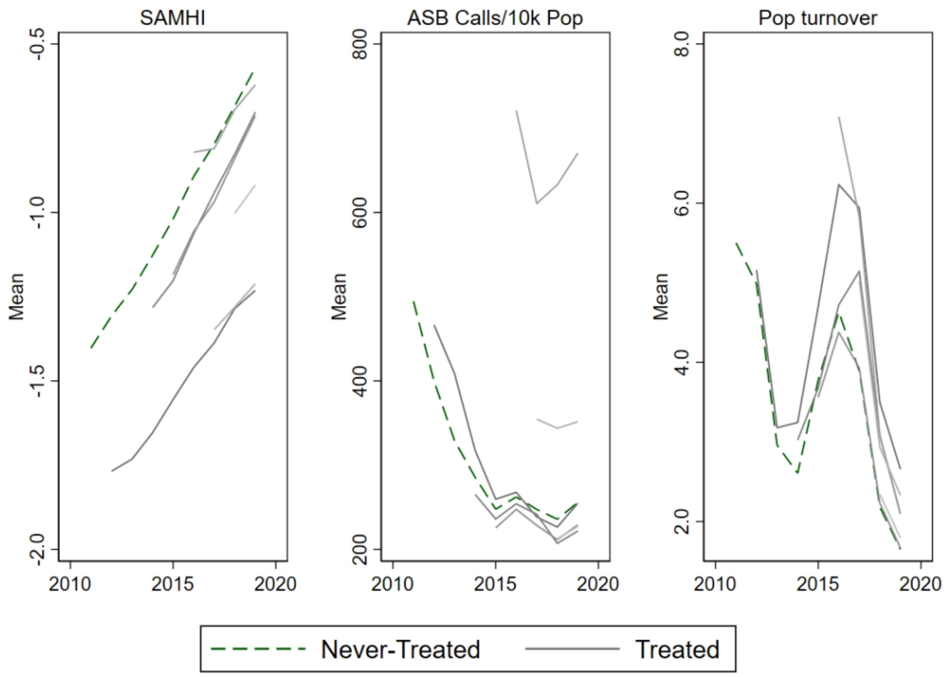

Trend in area-level outcomes for never-treated versus treated areas in Greater London, 2011-2019. Treated areas shown from year of initiation onwards. Abbreviations: Small Area Mental Health Index (SAMHI), Antisocial behaviour (ASB), Population (Pop).

423x307mm (72 x 72 DPI)

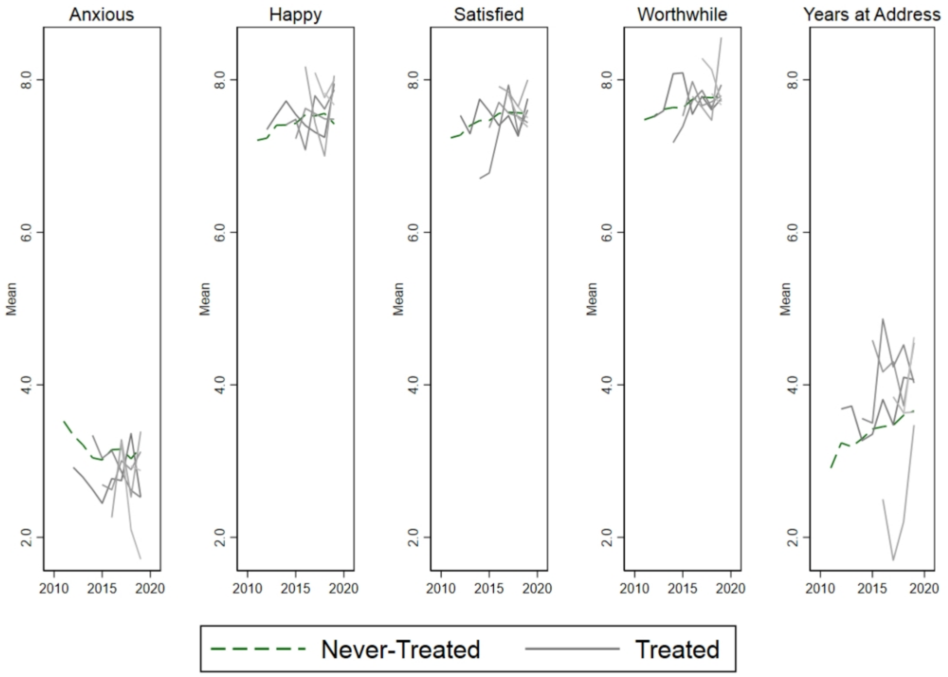

Trend in individual-level outcomes for never-treated versus treated areas. Treated areas shown from year of initiation onwards.

423x307mm (72 x 72 DPI)

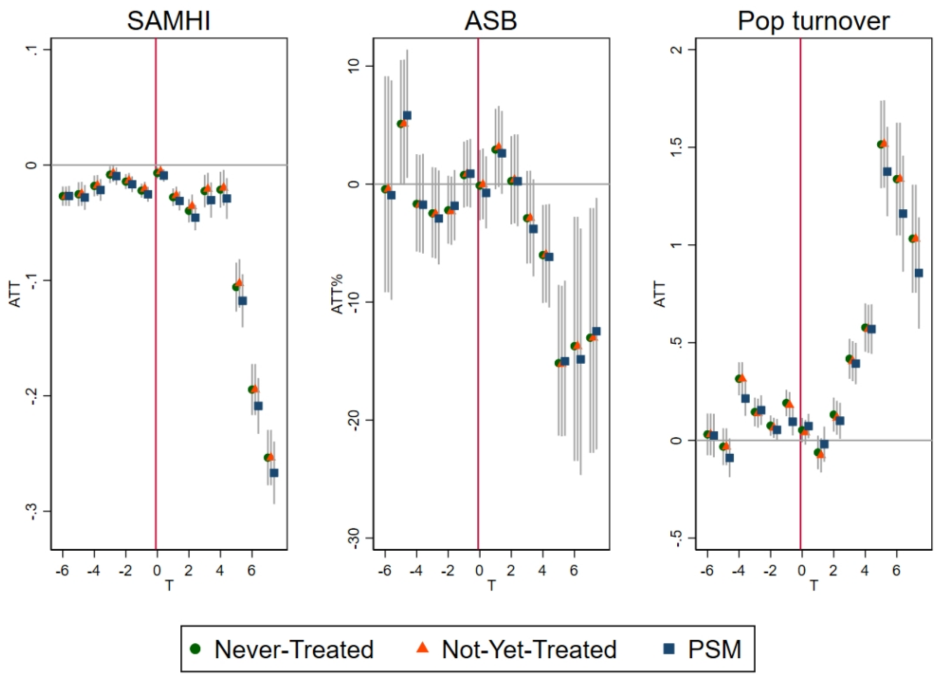

Average treatment effect on the treated (ATT) for area-level impacts of selective licencing (SL) on Small Area Mental Health Index (SAMHI), Antisocial behaviour (ASB) calls, and population (Pop) turnover in Greater London, 2011-2019. ASB was ln-transformed and ATT shown as ATT%.

423x307mm (72 x 72 DPI)

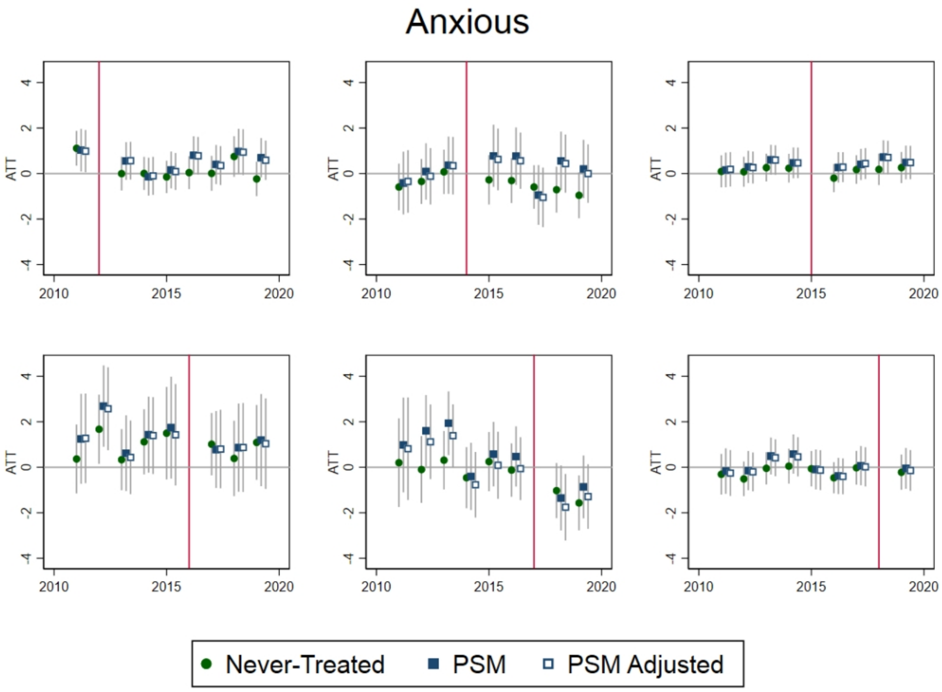

Average treatment effect on the treated (ATT) for individual-level impacts of selective licencing (SL) on self-reported anxiety among private renters in Greater London by year of SL introduction, 2011-2019. Time-varying covariates in PSM Adjusted were: age group, sex, native birth, and occupational class. Abbreviations: Propensity Score Matching (PSM).

423x307mm (72 x 72 DPI)

Supplementary materials

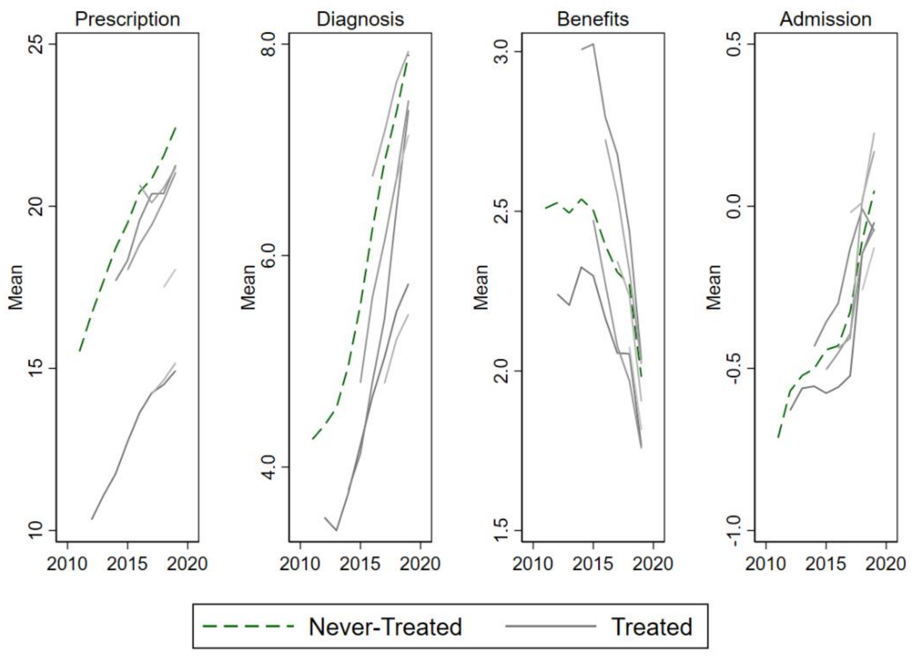

Supplementary Figure 1 Trend in Small Area Mental Health Index (SAMHI) sub-scores (PRESCRIPTION, DIAGNOSIS, BENEFITS, ADMISSION) for never-treated versus treated areas in Greater London, 2011-2019. Treated areas shown from year of initiation onwards..

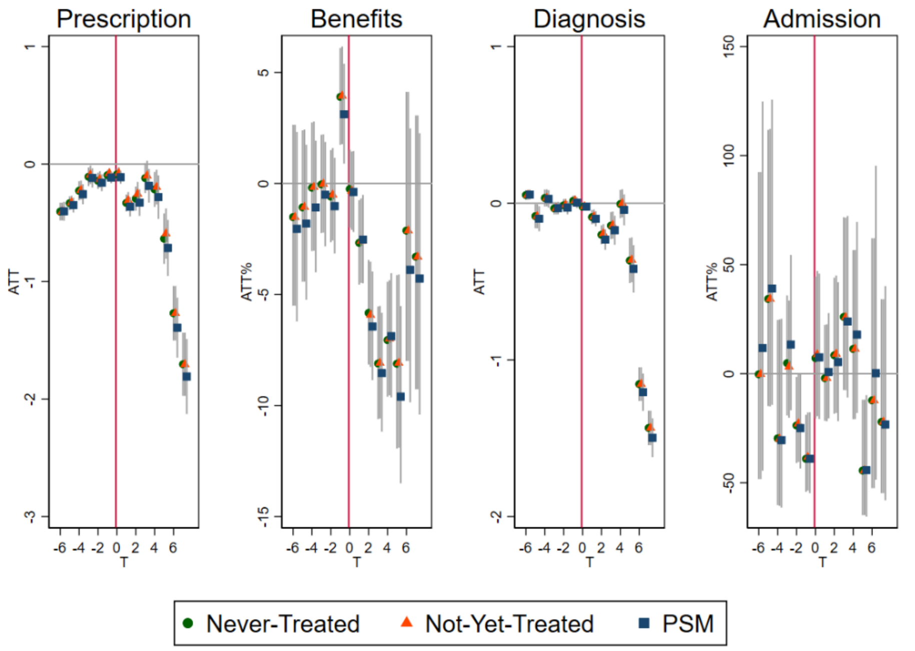

Supplementary Figure 2 Average treatment effect on the treated (ATT) for area-level impacts of selective licencing (SL) on Small Area Mental Health Index (SAMHI) underlying indicators PRESCRIPTION, BENEFITS, DIAGNOSIS, and ADMISSION, in Greater London, 2011-2019. BENEFITS and ADMISSION were ln-transformed and ATT shown as ATT%.

For peer review only - http://bmjopen.bmj.com/site/about/guidelines.xhtml

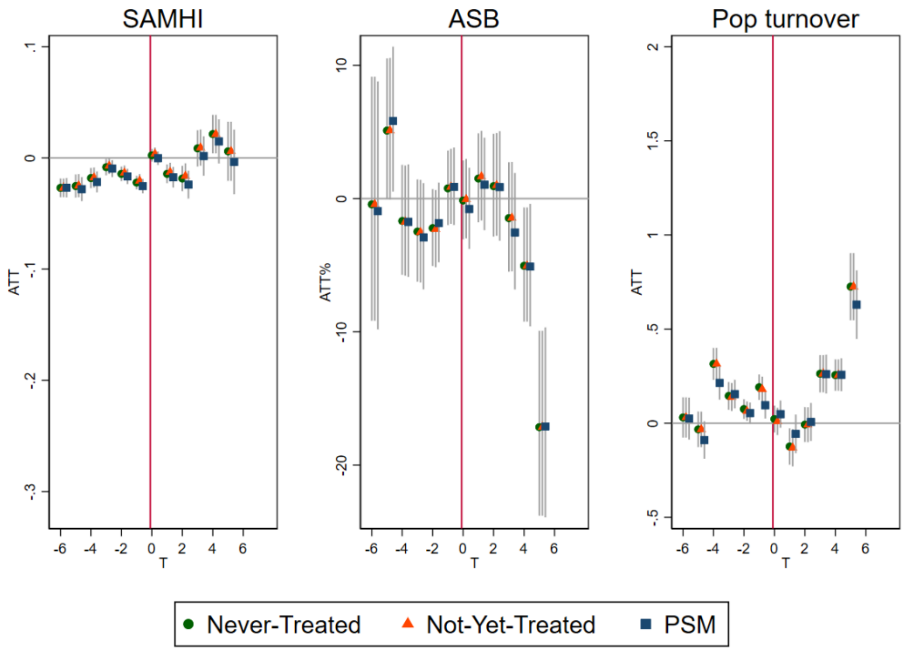

Supplementary Figure 3 Sensitivity check excluding the earliest scheme initiated 2012 ("Olympic"). Average treatment effect on the treated (ATT) for area-level impacts of selective licencing (SL) on Small Area Mental Health Index (SAMHI), Antisocial behaviour (ASB) calls, and population (Pop) turnover in Greater London, 2011-2019. ASB was ln-transformed and ATT shown as ATT%.

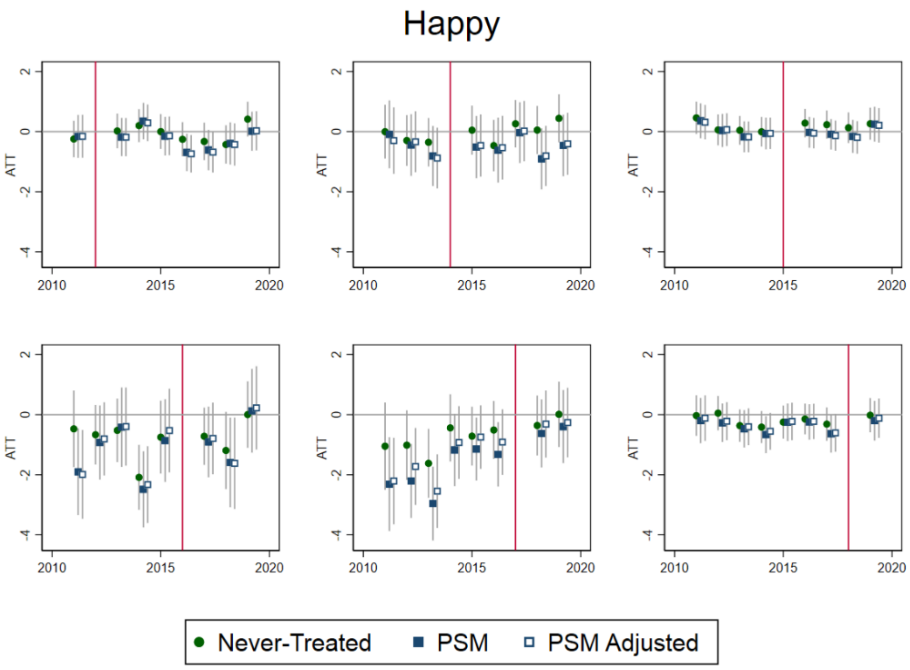

Supplementary Figure 4 Happy. Average treatment effect on the treated (ATT) for individual-level impacts of selective licencing (SL) on self-reported happy score among private renters in Greater London by year of SL introduction, 2011-2019. Time-varying covariates in PSM Adjusted were: age group, sex, native birth, and occupational class. Abbreviations: Propensity Score Matching (PSM).

For peer review only - http://bmjopen.bmj.com/site/about/guidelines.xhtml

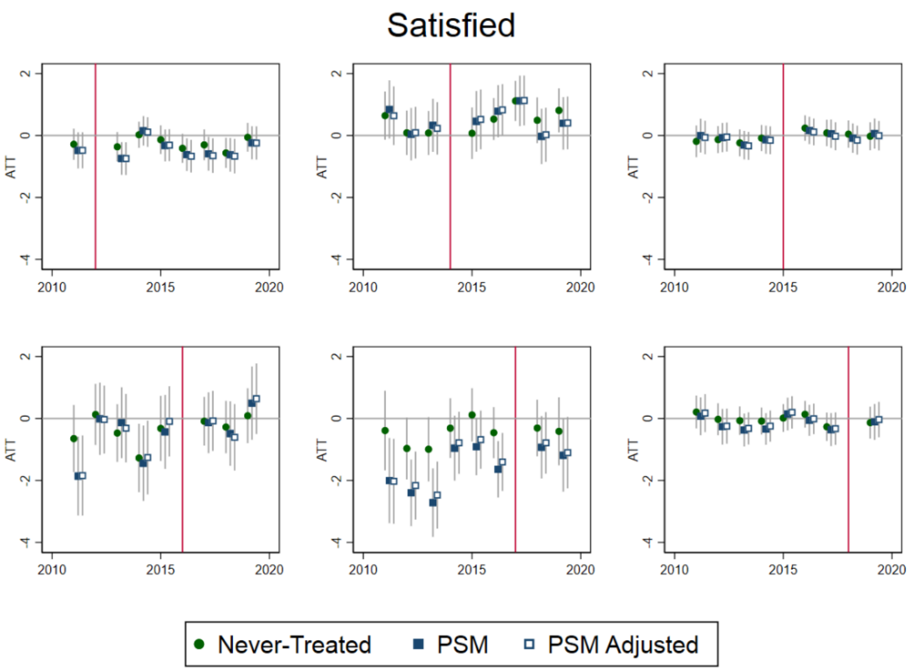

Supplementary Figure 5 Satisfied. Average treatment effect on the treated (ATT) for individual-level impacts of selective licencing (SL) on self-reported satisfied score among private renters in Greater London by year of SL introduction, 2011-2019. Time-varying covariates in PSM Adjusted were: age group, sex, native birth, and occupational class. Abbreviations: Propensity Score Matching (PSM).

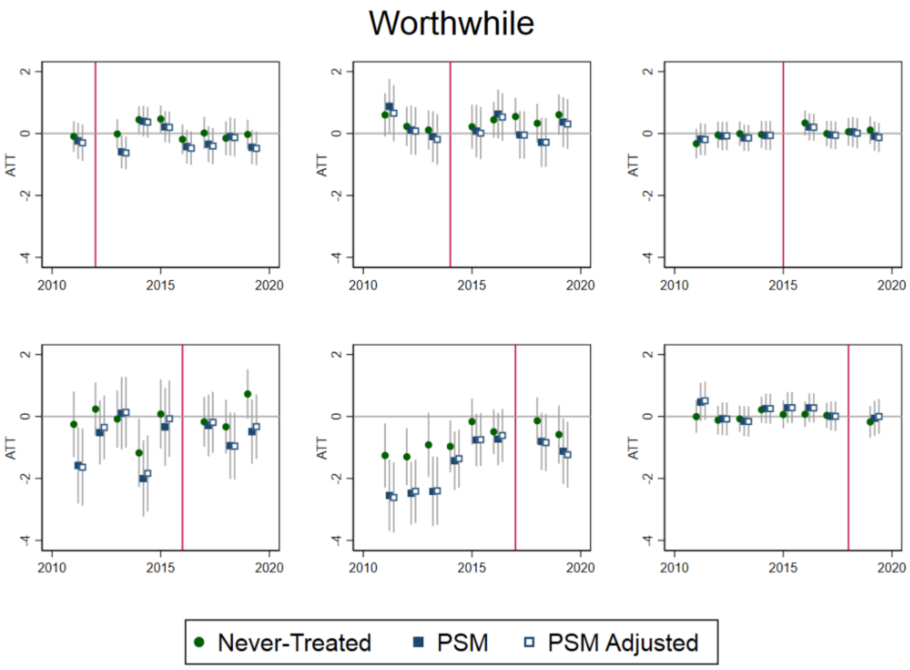

Supplementary Figure 6 Worthwhile. Average treatment effect on the treated (ATT) for individual-level impacts of selective licencing (SL) on self-reported worthwhile score among private renters in Greater London by year of SL introduction, 2011-2019. Time-varying covariates in PSM Adjusted were: age group, sex, native birth, and occupational class. Abbreviations: Propensity Score Matching (PSM).

For peer review only - http://bmjopen.bmj.com/site/about/guidelines.xhtml

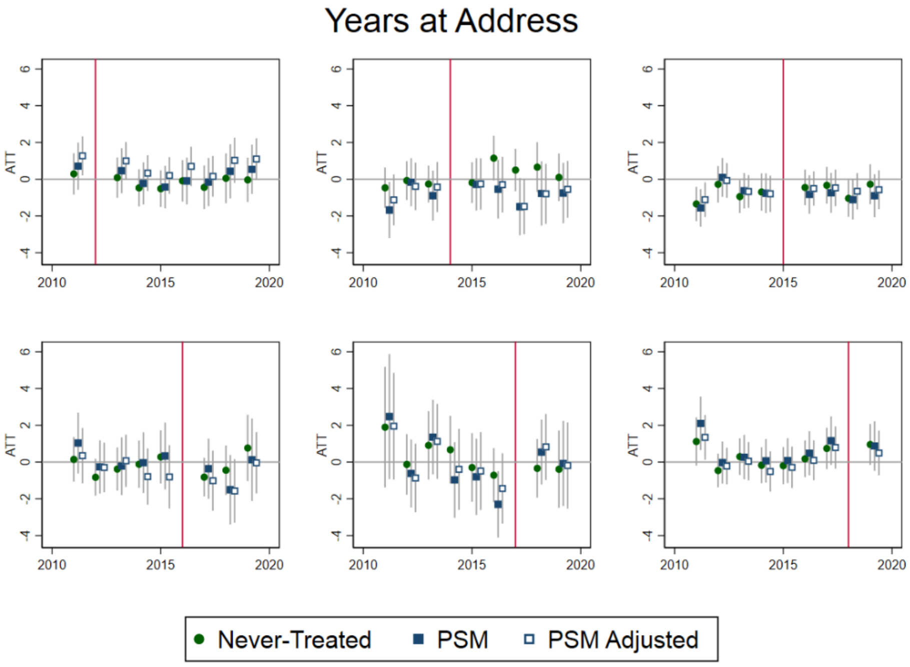

Supplementary Figure 7 Years at address. Average treatment effect on the treated (ATT) for individual-level impacts of selective licencing (SL) on self-reported years at address among private renters in Greater London by year of SL introduction, 2011-2019. Time-varying covariates in PSM Adjusted were: age group, sex, native birth, and occupational class. Abbreviations: Propensity Score Matching (PSM).

Supplementary Table 1 Baseline characteristics for Never-Treated and PSM control areas (LSOA) in Greater London, 2011. PSM controls were used for area-level impacts overall and for each year of treatment initiation, 2012 and 2014-2018, for individual-level impacts. Mean differences tested with a t-test except for Built pre-1945, which was tested with a Chi-square test (alpha=.05). Variables (Data source): Income deprived, Poor housing condition, No central heating, Unaffordable housing (Department for Communities and Local Government 2015b); Built pre-1945 (ONS 2021b); All other (ONS 2015a). Abbreviations: Lower Layer Super output Area (LSOA), Propensity Score Matched (PSM).

**Characteristics**	**All interventions**				
	**Treated**	**Never-Treated**		**PSM**	
	N=921	N=3,582	P-value	N=2,763	P-value
Children <16yr per pop	22.2	19.3	<.001	22.2	**0.839**
Adults 16-59yr per pop	64.1	64.3	**0.436**	63.8	**0.217**
Income deprived per pop	19.3	15.6	<.001	19.2	**0.823**
native birth per pop	60	65.3	<.001	60.6	**0.267**
Private rented%	25.9	23.4	<.001	25.3	**0.288**
Social rented%	22.7	22.5	**0.759**	22.8	**0.881**
Poor housing condition%	22.5	22.4	**0.621**	22.2	**0.298**
No central heating%	2.9	2.8	0.013	2.9	**0.906**
Overcrowded%	23.9	19.7	<.001	23.5	**0.402**
Unaffordable housing measure	2.4	1.7	<.001	2.4	**0.193**
Built pre-1945	-	-	<.001	-	**0.434**
**Characteristics**	**Interventions initiated in 2012**				
	**Treated**	**Never-Treated**		**PSM**	
	N=155	N=3,582	P-value	N=465	P-value
Children <16yr per pop	22.4	19.3	<.001	23.2	**0.153**
Adults 16-59yr per pop	68.2	64.3	<.001	67.7	**0.388**
Income deprived per pop	21.6	15.6	<.001	22.9	**0.15**
native birth per pop	46.5	65.3	<.001	47.4	**0.286**
Private rented%	33.9	23.4	<.001	33.4	**0.66**
Social rented%	28.9	22.5	<.001	31.9	**0.137**

Poor housing condition%	22.8	22.4	**0.368**	22.7	**0.763**
No central heating%	2.8	2.8	**0.807**	2.8	**0.906**
Overcrowded%	34.9	19.7	<.001	34.8	**0.946**
Unaffordable housing measure	2.8	1.7	<.001	2.9	**0.259**
Built pre-1945	-	-	**0.122**	-	**0.186**
**Characteristics**	**Interventions initiated in 2014**				
	**Treated**	**Never-Treated**		**PSM**	
	N=110	N=3,582	P-value	N=330	P-value
Children <16yr per pop	25.9	19.3	<.001	26.1	**0.753**
Adults 16-59yr per pop	59.9	64.3	<.001	59.9	**0.871**
Income deprived per pop	24.2	15.6	<.001	25.2	**0.354**
native birth per pop	69.5	65.3	0.003	69.2	**0.83**
Private rented%	17.5	23.4	<.001	16.7	**0.361**
Social rented%	33	22.5	<.001	34.8	**0.44**
Poor housing condition%	23.1	22.4	**0.221**	22.3	**0.115**
No central heating%	3	2.8	**0.126**	2.9	**0.49**
Overcrowded%	19.9	19.7	**0.9**	20	**0.86**
Unaffordable housing measure	2.7	1.7	<.001	2.74	**0.793**
Built pre-1945	-	-	**0.498**	-	**0.715**
**Characteristics**	**Interventions initiated in 2015**				
	**Treated**	**Never-Treated**		**PSM**	
	N=394	N=3,582	P-value	N=1,182	P-value
Children <16yr per pop	21.5	19.3	<.001	21.3	**0.468**
Adults 16-59yr per pop	62.8	64.3	<.001	62.2	**0.083**
Income deprived per pop	17.5	15.6	<.001	16.6	**0.073**
native birth per pop	65.6	65.3	**0.664**	67	**0.123**
Private rented%	22.6	23.4	**0.238**	21.3	**0.084**
Social rented%	19.7	22.5	0.008	18.4	**0.196**
Poor housing condition%	22	22.4	**0.25**	21.4	**0.079**
No central heating%	3	2.8	0.005	2.9	**0.255**
Overcrowded%	19.5	19.7	**0.7**	18.4	**0.11**
Unaffordable housing measure	2.2	1.7	<.001	2	**0.063**
Built pre-1945	-	-	0.001	-	**0.723**
**Characteristics**	**Interventions initiated in 2016**				
	**Treated**	**Never-Treated**		**PSM**	
	N=28	N=3,582	P-value	N=84	P-value
Children <16yr per pop	17.8	19.3	**0.106**	16.5	**0.319**
Adults 16-59yr per pop	72.4	64.3	<.001	74	**0.42**
Income deprived per pop	23.5	15.6	<.001	22	**0.443**
native birth per pop	53.6	65.3	<.001	52	**0.413**
Private rented%	33.5	23.4	<.001	36.6	**0.341**
Social rented%	33.3	22.5	0.005	28.8	**0.239**
Poor housing condition%	25.8	22.4	0.003	28	**0.227**
No central heating%	3.1	2.8	**0.226**	3.8	**0.184**
Overcrowded%	35.3	19.7	<.001	36.3	**0.705**
Unaffordable housing measure	3.2	1.7	<.001	2.8	**0.21**
Built pre-1945	-	-	0.024	-	**0.827**
**Characteristics**	**Interventions initiated in 2017**				
	**Treated**	**Never-Treated**		**PSM**	
	N=59	N=3,582	P-value	N=177	P-value
Children <16yr per pop	21.9	19.3	<.001	21.6	**0.79**
Adults 16-59yr per pop	65.5	64.3	**0.219**	66	**0.618**
Income deprived per pop	20.3	15.6	<.001	21.3	**0.371**
native birth per pop	45.4	65.3	<.001	45.5	**0.961**
Private rented%	33.2	23.4	<.001	35.1	**0.345**
Social rented%	18.6	22.5	**0.141**	20.1	**0.547**

For peer review only - http://bmjopen.bmj.com/site/about/guidelines.xhtml

Poor housing condition%	24	22.4	0.048	25.6	**0.148**
No central heating%	2.7	2.8	**0.564**	2.8	**0.701**
Overcrowded%	30.9	19.7	<.001	32.1	**0.407**
Unaffordable housing measure	3.5	1.7	<.001	3.3	**0.245**
Built pre-1945	-	-	0.027	-	**0.404**

**Characteristics**	**Interventions initiated in 2018**				
	**Treated**	**Never-Treated**		**PSM**	
	N=175	N=3,582	P-value	N=525	P-value
Children <16yr per pop	21.9	19.3	<.001	22.1	**0.806**
Adults 16-59yr per pop	64.3	64.3	**0.926**	64	**0.703**
Income deprived per pop	17.2	15.6	0.027	17	**0.787**
native birth per pop	58.8	65.3	<.001	58.8	**0.998**
Private rented%	27.8	23.4	<.001	27.5	**0.833**
Social rented%	17.2	22.5	<.001	17	**0.884**
Poor housing condition%	21.8	22.4	**0.266**	21.7	**0.708**
No central heating%	2.8	2.8	**0.866**	2.8	**0.747**
Overcrowded%	22.3	19.7	0.004	22	**0.785**
Unaffordable housing measure	2.2	1.7	<.001	2.2	**0.865**
Built pre-1945	-	-	0.001	-	**0.955**

For peer review only

STROBE Statement—Checklist of items that should be included in reports of ***cohort studies***

	Item No	Recommendation	Page No
**Title and abstract**	1	(*a*) Indicate the study's design with a commonly used term in the title or the abstract<br>(*b*) Provide in the abstract an informative and balanced summary of what was done and what was found	1-2
**Introduction**			
Background/rationale	2	Explain the scientific background and rationale for the investigation being reported	3
Objectives	3	State specific objectives, including any prespecified hypotheses	4
**Methods**			
Study design	4	Present key elements of study design early in the paper	4-5
Setting	5	Describe the setting, locations, and relevant dates, including periods of recruitment, exposure, follow-up, and data collection	4-5
Participants	6	(*a*) Give the eligibility criteria, and the sources and methods of selection of participants. Describe methods of follow-up<br>(*b*) For matched studies, give matching criteria and number of exposed and unexposed	4-5
Variables	7	Clearly define all outcomes, exposures, predictors, potential confounders, and effect modifiers. Give diagnostic criteria, if applicable	4-5
Data sources/ measurement	8*	For each variable of interest, give sources of data and details of methods of assessment (measurement). Describe comparability of assessment methods if there is more than one group	4-5
Bias	9	Describe any efforts to address potential sources of bias	6-8
Study size	10	Explain how the study size was arrived at	4-5
Quantitative variables	11	Explain how quantitative variables were handled in the analyses. If applicable, describe which groupings were chosen and why	4-5
Statistical methods	12	(*a*) Describe all statistical methods, including those used to control for confounding<br>(*b*) Describe any methods used to examine subgroups and interactions<br>(*c*) Explain how missing data were addressed<br>(*d*) If applicable, explain how loss to follow-up was addressed<br>(*e*) Describe any sensitivity analyses	4-5
**Results**			
Participants	13*	(a) Report numbers of individuals at each stage of study—eg numbers potentially eligible, examined for eligibility, confirmed eligible, included in the study, completing follow-up, and analysed<br>(b) Give reasons for non-participation at each stage<br>(c) Consider use of a flow diagram	5-6
Descriptive data	14*	(a) Give characteristics of study participants (eg demographic, clinical, social) and information on exposures and potential confounders<br>(b) Indicate number of participants with missing data for each variable of interest<br>(c) Summarise follow-up time (eg, average and total amount)	5-6
Outcome data	15*	Report numbers of outcome events or summary measures over time	5-6

For peer review only

Main results	16	(*a*) Give unadjusted estimates and, if applicable, confounder-adjusted estimates and their precision (eg, 95% confidence interval). Make clear which confounders were adjusted for and why they were included<br>(*b*) Report category boundaries when continuous variables were categorized<br>(*c*) If relevant, consider translating estimates of relative risk into absolute risk for a meaningful time period	5-6
Other analyses	17	Report other analyses done—eg analyses of subgroups and interactions, and sensitivity analyses	5-6
**Discussion**			
Key results	18	Summarise key results with reference to study objectives	6
Limitations	19	Discuss limitations of the study, taking into account sources of potential bias or imprecision. Discuss both direction and magnitude of any potential bias	6-8
Interpretation	20	Give a cautious overall interpretation of results considering objectives, limitations, multiplicity of analyses, results from similar studies, and other relevant evidence	6-8
Generalisability	21	Discuss the generalisability (external validity) of the study results	6-8
**Other information**			
Funding	22	Give the source of funding and the role of the funders for the present study and, if applicable, for the original study on which the present article is based	1

*Give information separately for exposed and unexposed groups.

Note: An Explanation and Elaboration article discusses each checklist item and gives methodological background and published examples of transparent reporting. The STROBE checklist is best used in conjunction with this article (freely available on the Web sites of PLoS Medicine at http://www.plosmedicine.org/, Annals of Internal Medicine at http://www.annals.org/, and Epidemiology at http://www.epidem.com/). Information on the STROBE Initiative is available at http://www.strobe-statement.org.

For peer review only
